# A tris(pyrazolyl)-based model for the aminocyclopropane carboxylic acid oxidase and its behavior towards oxidants

**DOI:** 10.1007/s00775-025-02125-w

**Published:** 2025-09-12

**Authors:** Lars Müller, Charikleia Tzatza, Santina Hoof, A. Jalila Simaan, Christian Limberg

**Affiliations:** 1 Institut für Chemie, Humboldt-Universität zu Berlin, Brook-Taylor-Straße 2, 12489 Berlin, Germany; 2 Aix Marseille Univ, Centrale Marseille, CNRS, iSm2 UMR 7313, 13397 Marseille, France

**Keywords:** Non-heme iron, Aminocyclopropane carboxylic acid oxidase, Dioxygen, Oxidation

## Abstract

A low-molecular-weight analog of the 1-aminocyclopropane carboxylic acid oxidase (ACCO), [Tp^Mes^FeACC], **1**, where the (His₂Asp)iron(II) moiety is mimicked by a hydrotris(3-mesitylpyrazol-1-yl)borato iron(II) unit, to which the natural substrate aminocyclopropane carboxylate is coordinated, has been accessed and structurally characterized. It was found to react slowly with O_2_ to yield the biological product ethylene. To create models of the intermediates proposed as part of the catalytic cycle of the ACCO **1** was treated with tBuOOH and mCPBA at low temperatures, which generated the respective Fe^III^OOtBu and Fe^IV^=O intermediates as shown by spectroscopic analysis. Studies on their behavior upon annealing reveal a non-biomimetic reactivity.

**Graphical Abstract:**

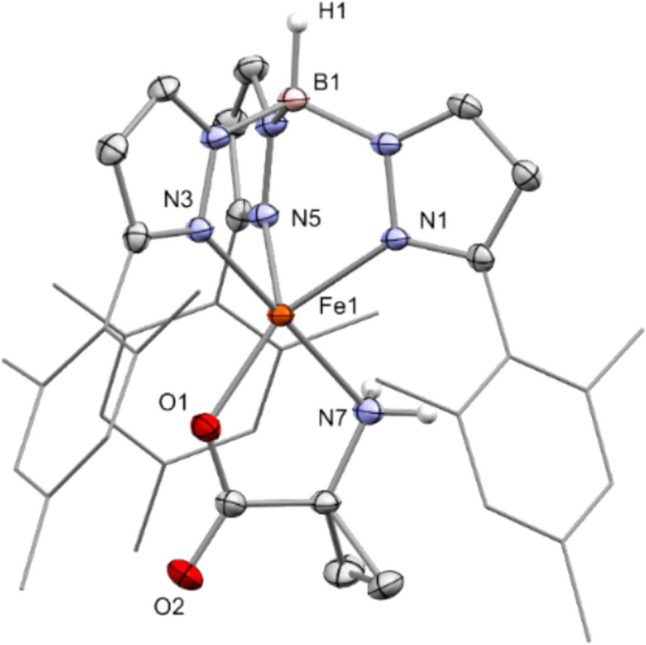

**Supplementary Information:**

The online version contains supplementary material available at 10.1007/s00775-025-02125-w.

## Introduction

**Scheme 1:**
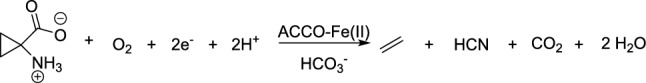
Biosynthesis of ethylene catalyzed by ACC oxidase (ACCO)

The enzyme 1-aminocyclopropane carboxylic acid oxidase (ACCO) catalyzes the oxidation of 1- aminocyclopropane carboxylic acid (ACCH) with molecular dioxygen to produce ethylene, hydrogen cyanide (HCN), carbon dioxide (CO_2_), and water. For this reaction, two equivalents of electrons and two equivalents of protons are required, which are possibly provided by ascorbic acid (Asc) (Scheme 1) [1–4].

**Fig. 1:**
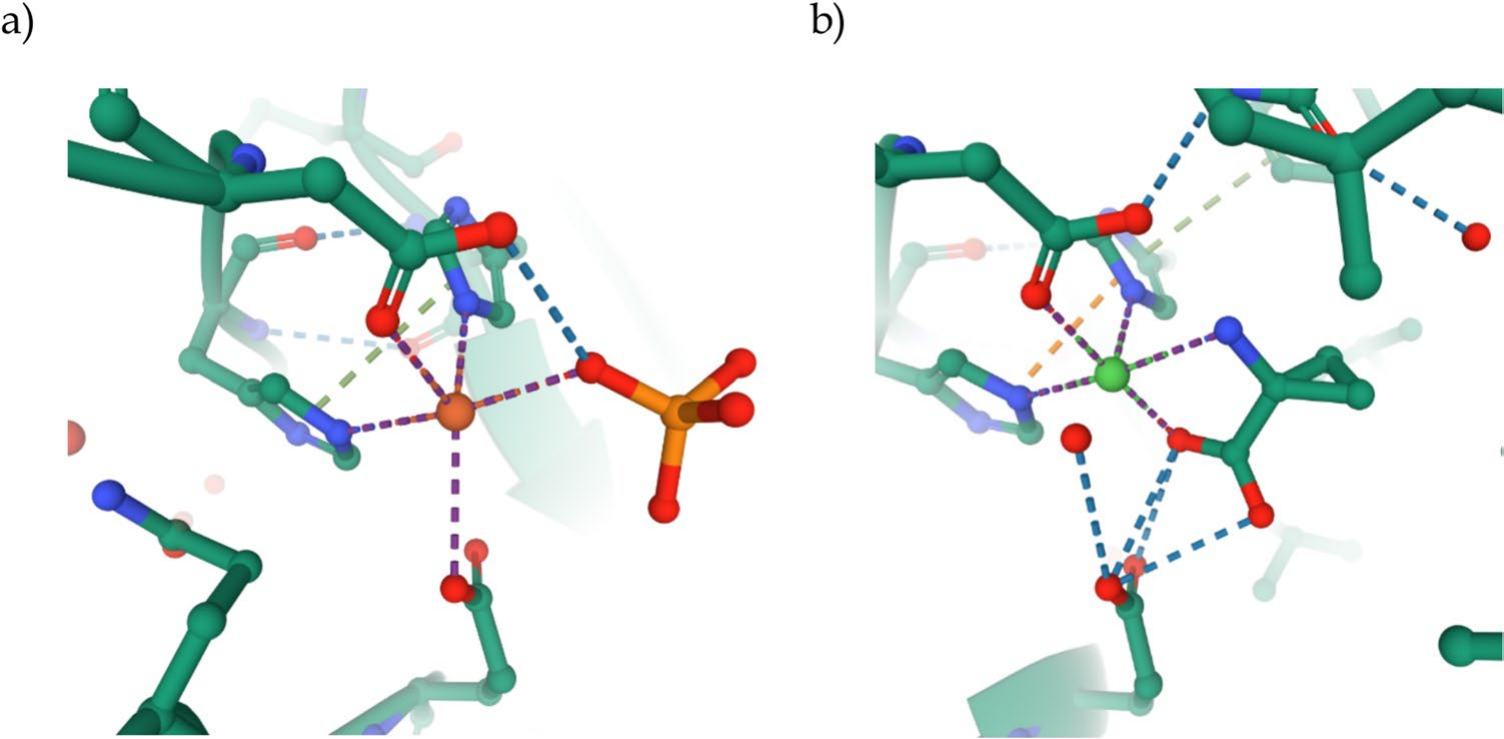
Active site of ACCO: a) without bound substrate [5] (PDB: 1WA6) and b) the nickel-substituted version with bound substrate (PDB: 5TCV); the graphics were created using Mol* [8]

Ethylene is one of the most important plant hormones, regulating processes such as root growth, fruit ripening, and senescence. The highly toxic cyanide produced in plants during ethylene biosynthesis is first converted into β-cyanoalanine and then further metabolized into asparagine for detoxification [1]. The active site of the enzyme consists of an iron(II) cofactor, which exhibits the facial His₂Asp coordination typical of non-heme oxidases. In the resting state, without a bound substrate, the iron ion is coordinated by two histidine residues, a carboxylate ligand from aspartic acid, and a phosphate ligand. A carboxylate residue from glutamic acid is also in close proximity but moves away from the metal center when the substrate coordinates with the iron [5]. This was confirmed by computational models by Simaan and coworkers [6] as well as by an X-ray crystallographic structure determination of a nickel-substituted enzyme–substrate complex [7] (Fig. 1).

**Scheme 2:**
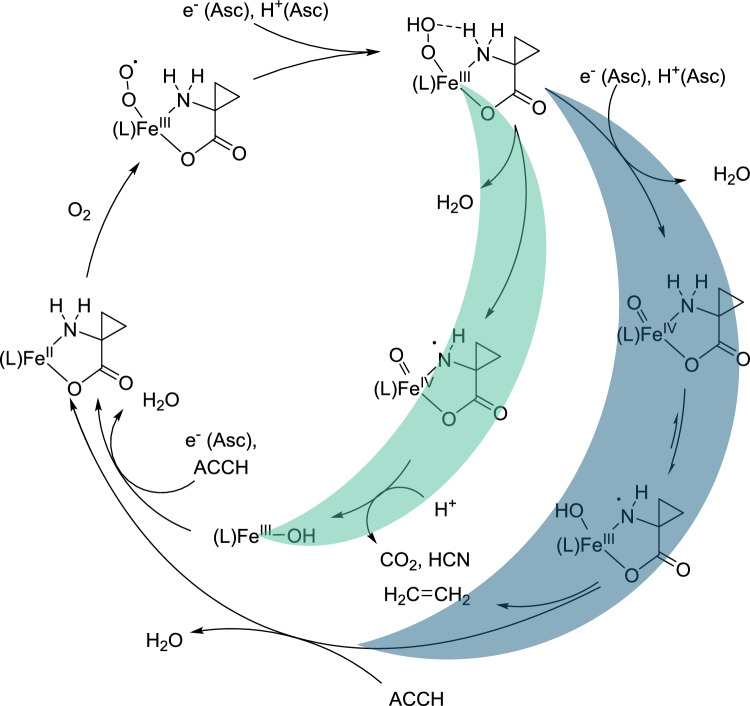
Proposed reaction mechanism of the ACCO (L = His_2_Asp), green: according to Lipscomb and coworkers [9], blue: according to Klinman and coworkers [10]

In this structure, the substrate binds to the metal in a *κ*^2^-*N,O* coordination mode. The role of a bicarbonate cofactor, which is essential for the activation of the enzyme, has not yet been fully clarified. Currently, two possible reaction mechanisms are under discussion (Scheme 2) [9, 10]. In both mechanisms, the reaction begins with the binding of dioxygen to the iron(II) center in its resting state, resulting in the formation of a superoxide species. This species is then reduced by ascorbic acid and subsequently protonated to form iron(III) hydroperoxide. According to the mechanism proposed by Lipscomb and colleagues, hydrogen atom abstraction occurs at the amine of the bound amino acid by the iron(III) hydroperoxide, followed by the homolytic cleavage of the O–O bond. The resulting iron(IV) oxo species, with a bound aminyl radical, decomposes in a cascade reaction to produce ethylene, hydrogen cyanide (HCN), carbon dioxide (CO₂), and an iron(III) hydroxo complex. The latter is then reduced and protonated by ascorbic acid. Finally, the coordination of a substrate ligand releases water and restores the enzyme to its resting state.

Alternatively, Klinman and colleagues propose that the intermediate iron(III) hydroperoxide is reduced and protonated a second time, leading to the release of water through heterolytic cleavage of the O–O bond, which generates an iron(IV) oxo species. This species then abstracts a hydrogen atom from the amine of the bound substrate, initiating the radical cascade that ultimately produces the products.

Hence, many mechanistic questions remain unanswered regarding the interaction mode with the substrate, the role of the different cofactors/co-substrates (ascorbic acid, dioxygen, and carbon dioxide) and the catalytic mechanism. Bioinorganic model studies can provide valuable information, both with regard to structural issues and requirements for reactivity. However, there are hardly any compounds known which may be regarded as models of ACCO, applying the minimum requirement that they contain ACC and that they display *substantial* ACCO-like activities. The following describes the chronological order of attempts to model the ACCO.

A first functional model was published in 2009 by Hitomi, Simaan, and coworkers. It is dinuclear; however, it contains *iron(III)* ions and *two* bridging ACC ligands as well as a µ-oxo bridge. Although this dinuclear iron(III) complex, **I**, is not a particularly good structural model for ACCO, it provides a moderate ethylene yield of 16% using hydrogen peroxide as the oxidant and two to three equivalents of sodium hydroxide. Reaction intermediates in these reactions were also not observed even at low temperatures [11].

Sometime later, Simaan, Mahy, and colleagues published the iron(II) complex [Fe(BPMEN)ACC]SbF₆, **II**, based on the N,N′-dimethyl-N,N′-bis(pyridylmethyl)ethane-1,2-diamine ligand (BPMEN). This compound demonstrated for the first time the *κ*^2^-*N,O* binding mode of the substrate ligand as it is assumed for the enzyme. However, **II** can hardly be considered a functional replicate of the ACCO, as the iron center is coordinated octahedrally and hence oxidants have no access to the iron center. This complex could also react with hydrogen peroxide, yielding ethylene amounts of up to 23%, which is, however, only 7% higher compared to the blank experiments. A reaction with dioxygen was not possible [12].

**Scheme 3:**
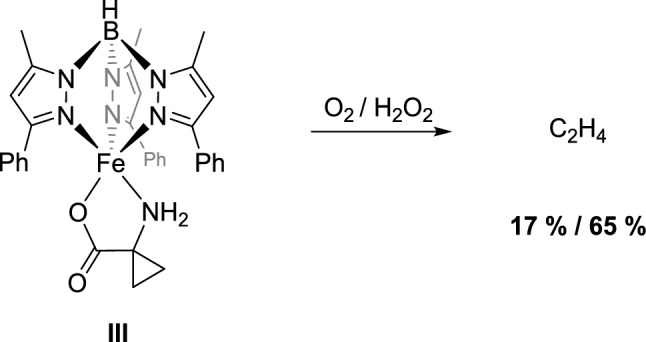
Structural and functional ACCO model [Tp.^Ph,Me^FeACC], **III**, by Limberg, Simaan, and coworkers [13]

Shortly thereafter, Limberg, Simaan, and colleagues published a model system based on the Tp^Ph,Me^ ligand, [Tp^Ph,Me^FeACC], **III**, which features a five-coordinate iron center and thus provides a free coordination site for the binding of oxygen or hydrogen peroxide (Scheme 3) [13]. **III** is not only an excellent structural model for ACC oxidase, where the substrate ligand coordinates to the iron(II) atom in a *κ*^2^-*N,O* binding mode, but it also serves as a very good functional model for ACCO. Using hydrogen peroxide, an ethylene yield of 65% is achieved, and a reaction with oxygen produces up to 17% ethylene. Unfortunately, the molecular structure of the compound could not be determined via single-crystal X-ray diffraction, as single crystals were only available from the nickel analog, [Tp^Ph,Me^NiACC]. The identification of reaction intermediates or products other than ethylene was also not possible.

**Scheme 4:**
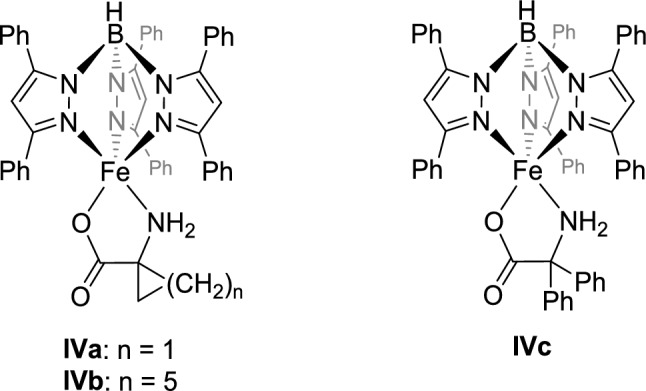
Model compounds [Tp^Ph2^FeACC], **IVa**, [Tp^Ph2^FeACH], **IVb**, and [Tp.^Ph2^FeADP], **IVc**, by Paine and coworkers [14]

Subsequently, Paine and coworkers successfully synthesized another model based on a Tp ligand, [Tp^Ph2^FeACC], **IVa** (Scheme 4), and structurally characterized it using X-ray diffraction experiments. The colorless high-spin iron(II) complex exhibits a distorted square pyramidal coordination (τ₅ = 0.42). Additionally, the two complexes [Tp^Ph2^FeACH], **IVb**, and [Tp^Ph2^FeADP], **IVc**, were synthesized and thoroughly characterized using 1-aminocyclohexane carboxylic acid (ACHH) and 2-amino-2,2-diphenylacetic acid (ADPH)**.** The reaction of **IVa** with oxygen at 70 °C results in conversions of up to 28%, with ethylene and carbon dioxide anticipated as the sole products. The substrate ligands of **IVb** and **IVc** are converted at 70 °C into cyclohexanone (15%) and acetophenone (45%), respectively. No intermediates could be detected in these reactions, leaving the mechanistic pathway of the reaction still unresolved [14].

In previous work concerning the modeling of the cysteine dioxygenase with the aid of Tp-Fe complexes, we noted that a seemingly minor change, namely, formally exchanging phenyl residues at the pyrazolyl donors for mesityl residues brought about major advantages: due to the increased steric bulk the previously unstable product complex after oxygenation could be isolated and structurally characterized and at the same time the reaction went 200 times faster, as—counterintuitively—the reaction pocket was more spacious [15]. Hence, we planned to test this “mesityl effect” also for **III** and exchange phenyl for mesityl, hoping that this may lead to a structural characterization of the ACC complex and the investigation of reaction intermediates. Here we report the results.

## Results and discussion

### Synthesis and characterization of the ACCO model

**Scheme 5:**
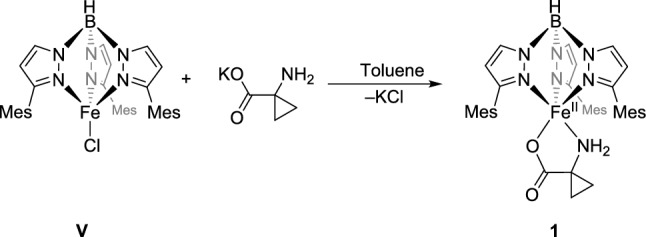
Synthesis of the model complex [Tp^Mes^FeACC], **1**, starting from the precursor **V** and KACC

**Fig. 2:**
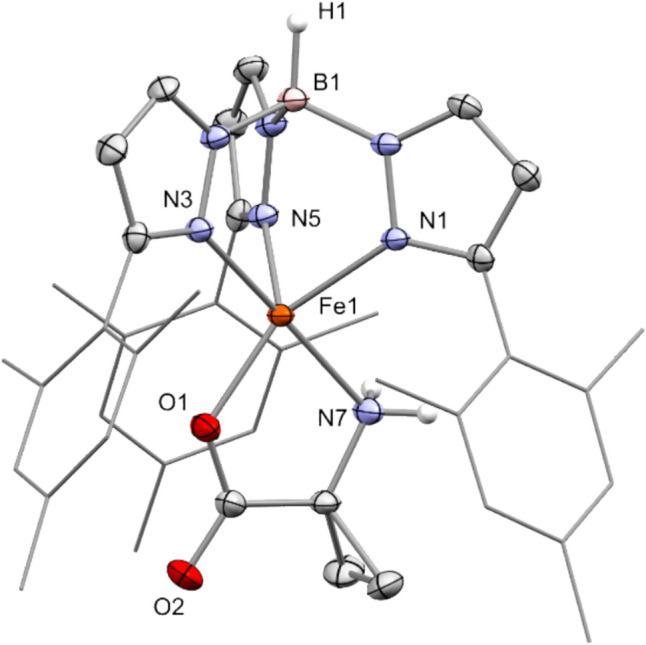
Molecular structure of [Tp.^Mes^FeACC], **1**·0.5C_6_H_6_, as determined by single crystal X-ray diffraction. Hydrogen atoms, except for those at the boron and nitrogen atoms, as well as non-coordinating solvent molecules, have been omitted for clarity. Selected bond lengths (in Å) and angles (in °): Fe1-N1 = 2.1099(16), Fe1-N3 = 2.1887(16), Fe1-N5 = 2.1146(16), Fe1-N7 = 2.2211(17), Fe1-O1 = 1.9499(14), N3-Fe1-N7 = 173.26(7), O1-Fe1-N1 = 148.43(6)

Starting from the precursor complex **V** and potassium 1-aminocyclopropanecarboxylate (KACC), the target complex [Tp^Mes^FeACC], **1,** was synthesized in a metathesis reaction with a good yield of 74% and isolated as an almost colorless compound (Scheme 5). The complex crystallizes with non-coordinating benzene solvent molecules in the form of **1**·0.5C₆H₆ (Fig. 2).

The result of the X-ray structural analysis confirms that [Tp^Mes^FeACC], **1**, is a mononuclear complex in which the iron center is facially coordinated by three nitrogen atoms of the Tp ligand, coordinating in a *κ*^3^-binding mode, as well as one oxygen atom and one nitrogen atom from the amino acid in a *κ*^2^-*N*,*O*-coordination, which is also postulated for the enzyme [6]. The C–O bond lengths of the carboxylate function are significantly different, measuring 2.29 and 2.22 Å, indicating a localization of the negative partial charge on the coordinating oxygen atom. The structural parameter **τ₅** of 0.41 compares well with that of the model **IVa** (0.42) and also with the value determined for the calculated structure of the ACCO (0.47) [6], while that of the abovementioned nickel-substituted enzyme–substrate complex[7] is somewhat higher (0.77). It thus lies between that of a square pyramid and a trigonal bipyramid, making the sixth free coordination site readily accessible.

**Fig. 3:**
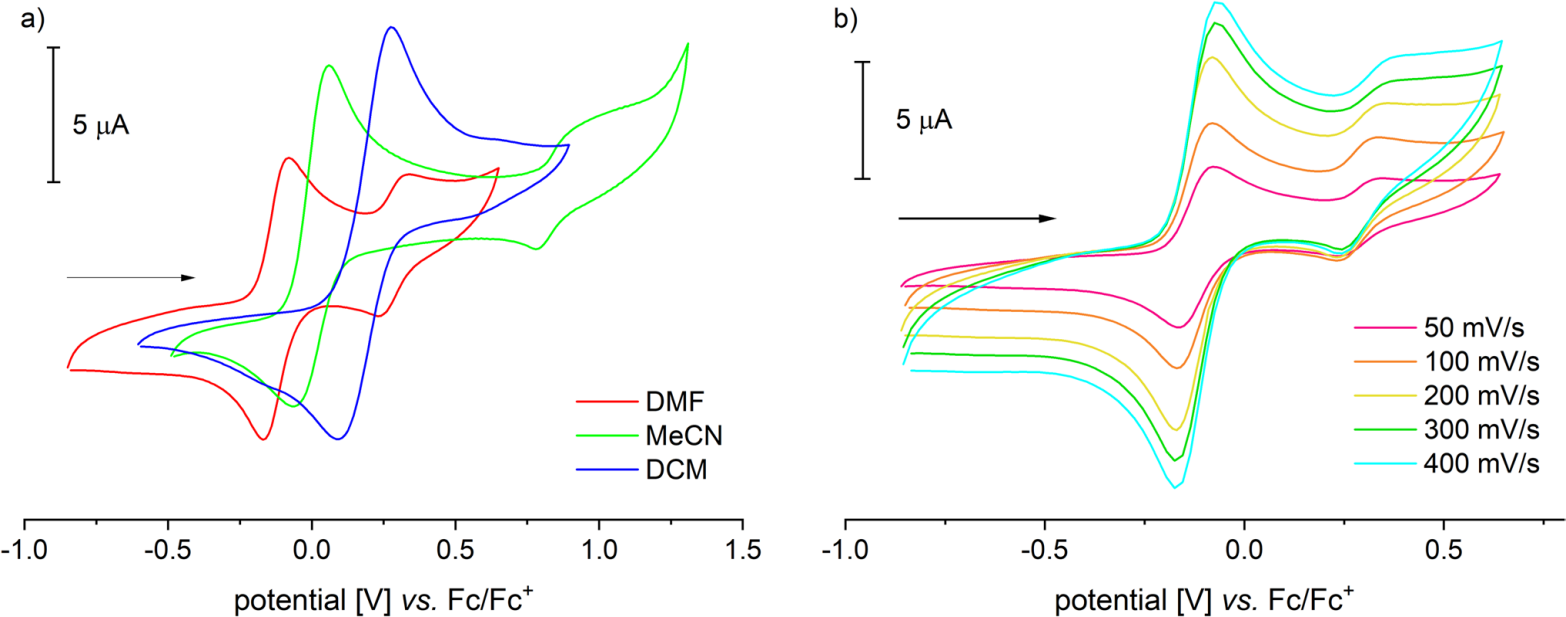
Cyclic voltammograms of [Tp.^Mes^FeACC], **1**, **a** measured in DMF, MeCN and DCM with scan rates of 100 mV/s and **b** in DMF with scan rates between 50 and 400 mV/s (RT, 100 mM TBAF, 1 mM complex)

The compound exhibits well-resolved and paramagnetically shifted signals in the ^1^H-NMR spectrum in the range of − 15 to 55 ppm. ATR-IR spectra clearly show the characteristic absorptions of the NH₂ group for Tp-based ACCO models at 3353 and 3288 cm⁻^1^ (**III**: 3360, 3304; **IVa**: 3445, 3366 cm⁻^1^), the B–H vibration at 2474 cm⁻^1^ (**III**: 2549, **IVa**: 2625 cm⁻^1^), and the antisymmetric carboxylate vibration at a wavenumber of 1658 cm⁻^1^ (**III**: 1657, **IVa**: 1639 cm⁻^1^) [13, 14]. High-resolution mass spectrometric investigations on **1** revealed a signal with a mass-to-charge ratio of 724.341 m/z, corresponding to that of the singly protonated molecular ion (MH⁺). The Mössbauer spectrum of **1**, recorded from the solid sample at 14 K, displays a doublet with an isomer shift *δ* = 1.08 mm/s and a quadrupole splitting of ΔEq = 2.35 mm/s. These values are consistent with a high-spin iron(II) species and are comparable to those determined for **III** (*δ* = 1.09 mm/s, ΔEq = 2.7 mm/s) [13]. Additionally, **1** was electrochemically investigated using cyclic voltammetry. Voltammograms of DMF, MeCN, and DCM solutions were recorded (Fig. 3). The compound exhibits reversible redox events in all three solvents used, which have been attributed to the Fe^2^⁺/Fe^3^⁺ redox couple. The position of the half-wave potential is strongly dependent on the donor properties of the solvent employed. In the most polar solvent, DMF, *E*_1/2_ is – 0.125 V (*E*_pa_ = – 0.080 V, *E*_pc_ = – 0.170 V). In acetonitrile, it is at 0 V (*E*_pa_ =  + 0.055 V, *E*_pc_ = – 0.055 V), and in dichloromethane, it is + 0.185 V (*E*_pa_ =  + 0.290 V, *E*_pc_ =  + 0.080 V), each measured against Fc/Fc⁺ (Fig. 3).

The significant dependence of the redox potential on the solvent used suggests coordination of the respective solvent, DMF or MeCN, at the iron center, which results in a shift of the redox potential compared to the potential without additional coordination (in DCM). The higher the basicity of the solvent, the greater the shift in potential. Thus, it can be assumed that **1** exists in the coordinating solvents as the adduct complexes [Tp^Mes^FeACC(DMF)], **1(DMF)**, and [Tp^Mes^FeACC(NCMe)], **1(MeCN)**.

The data presented above indicates that [Tp^Mes^FeACC], **1**, is more challenging to oxidize under comparable conditions than the previously studied model complex [Tp^Ph,Me^FeACC], **III**, which was non-reversibly oxidized at 0.03 V in dichloromethane [13]. Another distinction arises from the cup-like arrangement of the mesityl substituents, which protects the oxidized form of **1** from subsequent reactions, making it possible to reliably determine E_1/2_ of the (quasi)reversible redox event. Assuming that the diffusion coefficients of the oxidized and reduced species are identical, E_1/2_ corresponds to the standard potential E°. In the case of an irreversible redox event, a significant shift in the peak potentials is often observed, as there is no thermodynamic equilibrium; therefore, the Nernst equation is no longer applicable [16]. Therefore, a direct comparison of the redox potentials of **1** and **III** is only partially meaningful, and conclusions about the reactivity of the compounds toward oxygen cannot be drawn based on the redox potentials.

**Fig. 4:**
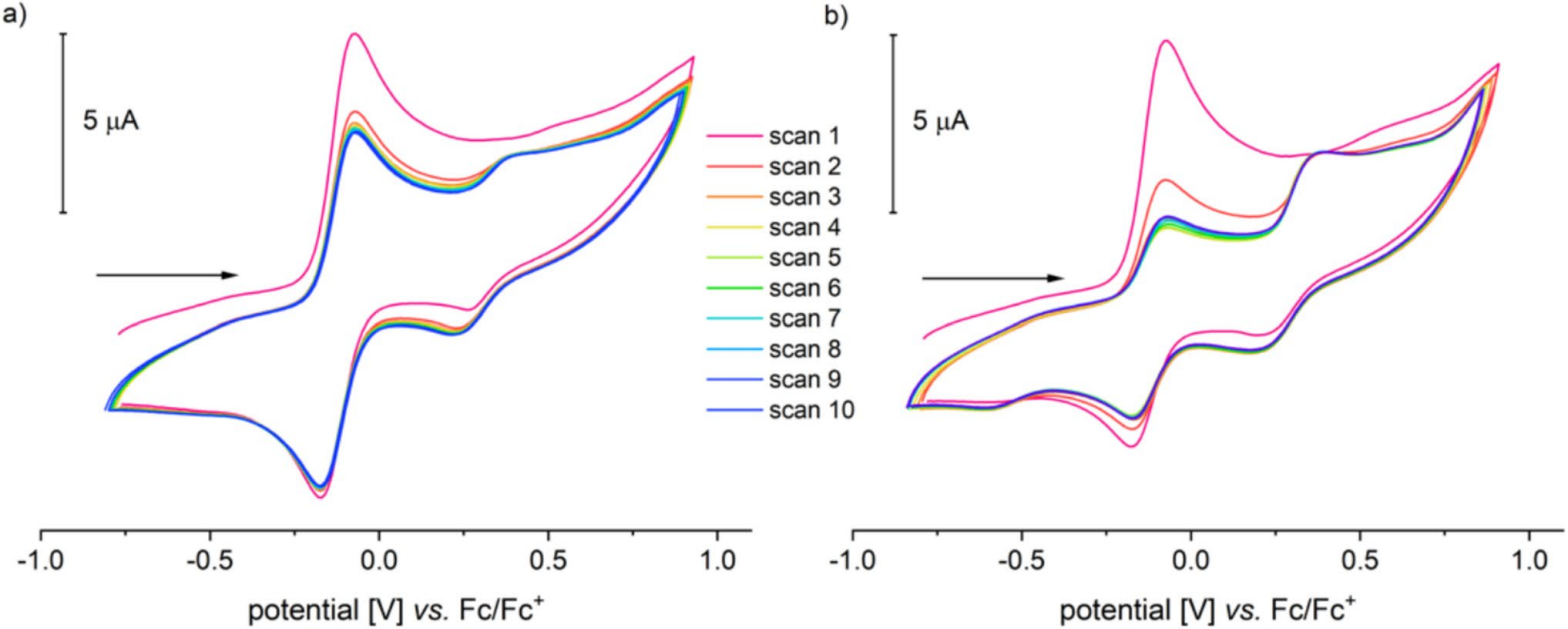
Cyclic voltammograms of [Tp.^Mes^FeACC], **1**, **a** in dry DMF and **b** with 100 equivalents of water added (RT, 0.1 V/s, 100 mM TBAF, 1 mM complex)

Notably, only in DMF does another quasi-reversible redox event appear in the cyclic voltammogram of compound **1**, with a half-wave potential of 0.335 V (*E*_pa2_ = 0.380 V, *E*_pc2_ = 0.290 V). The ratio of the current of the first redox event to that of the second changes little with increasing scan rate, which argues against a consecutive redox event. Moreover, the second redox event does not appear in the first scan, which also contradicts a classical ECE mechanism (ECE = electrochemical, chemical, electrochemical). In such a case, the second redox event would be coupled to the first but independent of the number of measurement cycles [16, 17]. Additionally, it is noticeable that the reduction of the second redox process can be observed already in the first scan, without any preceding oxidation at a higher potential. This implies a reaction of the previously oxidized species, which forms a more easily reducible species. As the number of scans increases, the current of the second event rises. This effect is further amplified when a small amount of water is added to the solution (Fig. 4a, b).

**Scheme 6:**
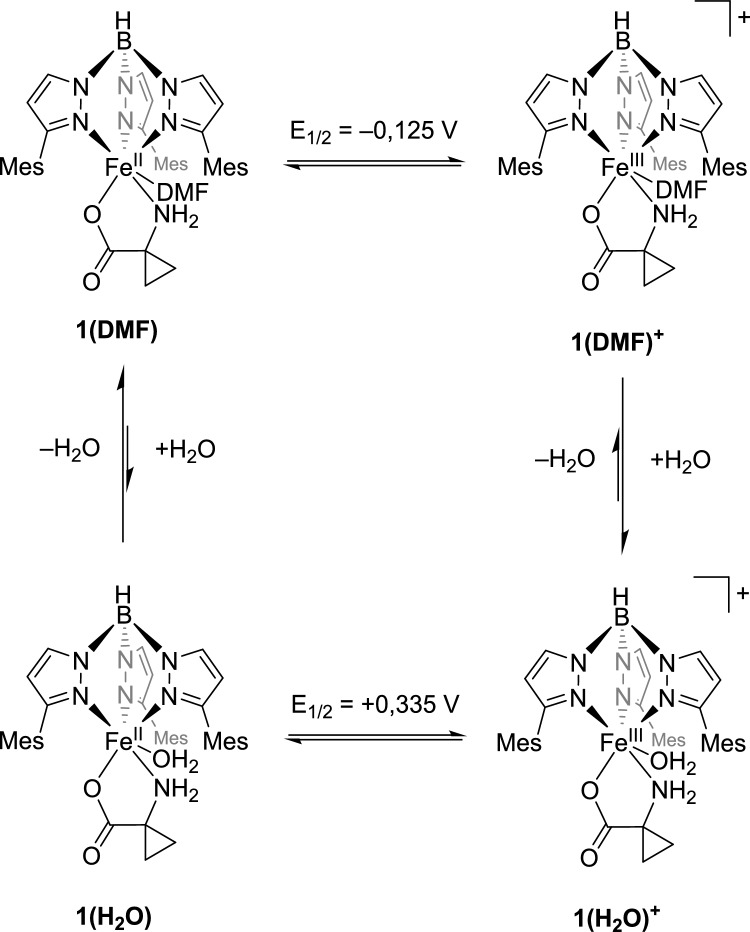
Possible explanation for the second redox event at 0.335 V versus Fc/Fc^+^ in the cyclic voltammogram of [Tp^Mes^FeACC], **1**, in DMF by the exchange of a DMF ligand for an aqua ligand

A plausible scenario to explain the observations is the coordination of a weak aqua ligand instead of the DMF ligand at the free coordination site of [Tp^Mes^FeACC], **1**. This results in a classical square scheme that links two coupled EC reactions (Scheme 6) [18]. If the potential had changed due to the protonation of **1** by the weak acid water, the cyclic voltammogram would exhibit a different characteristic shape. Here, a typical CE wave would appear with a plateau at the initially observed potential, followed by a subsequent oxidation wave at a higher potential, where an equilibrium reaction precedes the electron transfer [18, 19].

A prerequisite for the observations is the different equilibrium positions between **1(DMF)** and **1(H**_**2**_**O)** in the reduced and oxidized states; in the oxidized state, coordination with H_2_O appears to be favored. The slow rate of ligand exchange is another condition for the observations made here. In the case of very fast ligand exchange, there would not be an accumulation of **1(H**_**2**_**O)**, and consequently, a second oxidation wave would not occur. A possible reason why the second redox event was observed exclusively in DMF is the difficulty of completely drying this solvent. Although the solvent used for CV experiments was stored over molecular sieves, it may have still contained traces of water, which could influence the results.

Exclusively in acetonitrile, alongside the reversible Fe^II^/Fe^III^ redox event, another irreversible anodic process appears at around 1 V, which is not observed in DMF and DCM solutions because it is already outside the stability range of these solvents. This electron transfer can be attributed to the oxidation of the coordinated ACC substrate ligand.

### Dioxygen activation

At room temperature, **1** shows only a very slow reaction with oxygen, and no reaction can be observed at low temperatures. This sluggish reactivity can be attributed to its comparatively high oxidation potential, which is significantly above − 0.1 V against Fc/Fc^+^. Studies were conducted on the reactivity of **1** with oxygen, investigating the production of ethylene. While ethylene was detected in these studies (conversion of 4–5% of the bound ACC into ethylene), it was not produced to a greater extent than that observed for the preceding model [Tp^Ph,Me^FeACC], **III**.

Since it had been previously shown that the Tp^Mes^ ligand provides more space for the coordination and reactivity of oxygen than the Tp^Ph,Me^ ligand, it can be assumed that the moderate reactivity of both **1** and **III** is not due to a lack of accessibility of the iron center but has electronic reasons. Superoxide formation is often endergonic, and comparatively positive redox potentials are not helpful; superoxide dissociation competes and thus also other side reactions. Therefore, elucidating the reaction mechanism through studies with **1** and molecular oxygen did not appear promising, and reduced oxygen species were employed instead (“shunt routes”) to enter the turnover cycle at later stages and potentially observe intermediates.

### Reactivity towards ^t^BuOOH

The use of reduced oxygen species, such as hydroperoxides, offers the advantage of bypassing the initial steps in the enzymatic reaction. These steps typically involve the binding of oxygen to the metal, leading to the formation of an iron(III) superoxide species, which is then reduced and protonated to yield an iron(III) hydroperoxide. By using reduced oxygen species, an iron(III) peroxo species can be generated directly. The latter may then, as proposed by Lipscomb and colleagues, react further via a radical cascade that ultimately leads to the formation of the products ethylene, HCN, CO_2_, and water [9]. Alternatively, an O–O bond cleavage could be envisioned, which would generate a high-valent iron(IV)-oxo species. This species, similar to the mechanism proposed by Klinman and colleagues, could initiate the radical cascade that ultimately produces the reaction products [10]. H_2_O_2_ is a possible source of peroxide but has two OH units that may open up various reaction routes. We therefore decided to investigate the reactivity of **1** with ^*t*^BuOOH; potentially formed FeOO^*t*^Bu units would model the FeOOH species in Fig. 2 and produce alcohol instead of water then.

**Fig. 5:**
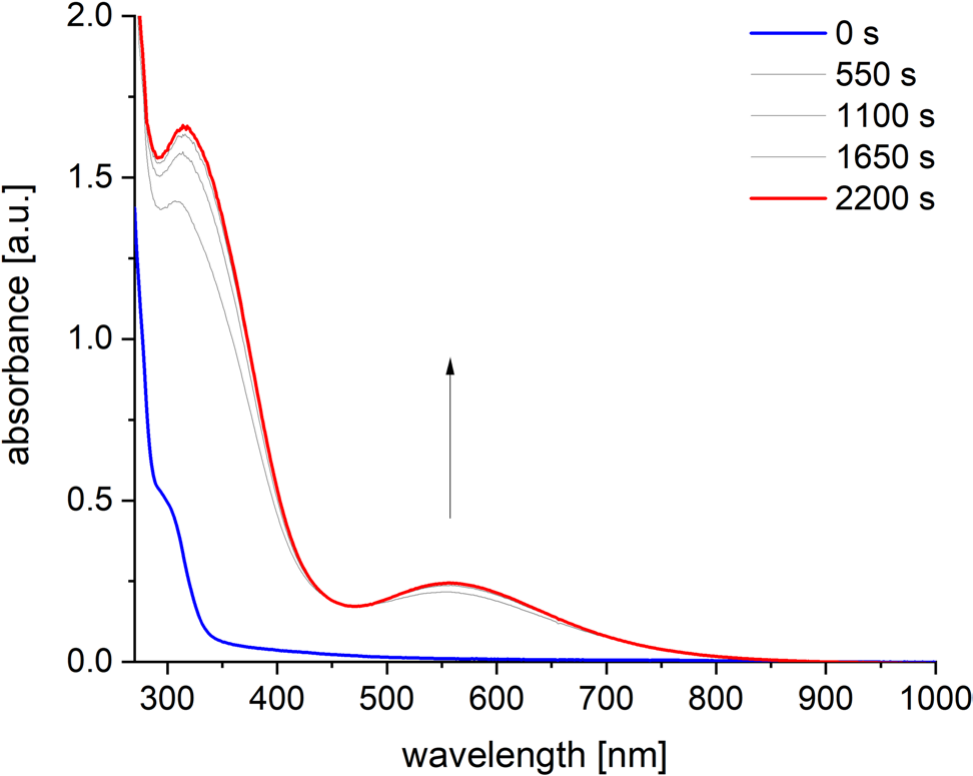
UV–Vis spectrum of [Tp^Mes^Fe(OO^*t*^Bu)(ACC)], **2**, generated from **1** through treatment with four equiv. of ^*t*^BuOOH at – 80 °C in DCM (0.4 mM)

By analogy to previous investigations on the reactivity of [Tp^Mes^FeO_benzoate_] complexes [20] the formation of a temperature-sensitive, intensely violet-colored species was observed as soon as the alkyl peroxide was added in slight excess to the solution. The UV–Vis spectrum of this species exhibits two absorption maxima at 315 and 560 nm (Fig. 5). The band at 560 nm can be attributed, as previously in the case of [Tp^Mes^Fe(OO^*t*^Bu)(OBz^*R*^)] compounds [20], to a π* → *d* transition.

**Scheme 7:**
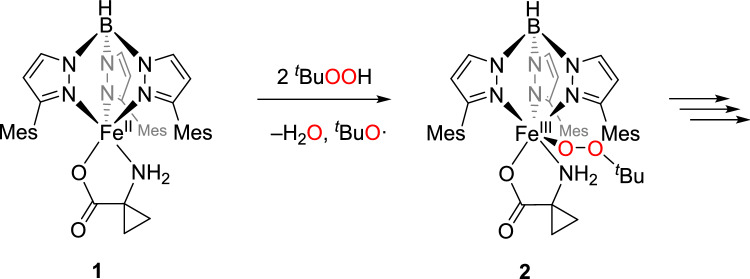
Generation of the iron(III)alkylperoxide [Tp^Mes^Fe(OO^*t*^Bu)(ACC)], **2**, through the reaction of [Tp^Mes^FeACC], **1**, with an excess of ^*t*^BuOOH

**Fig. 6:**
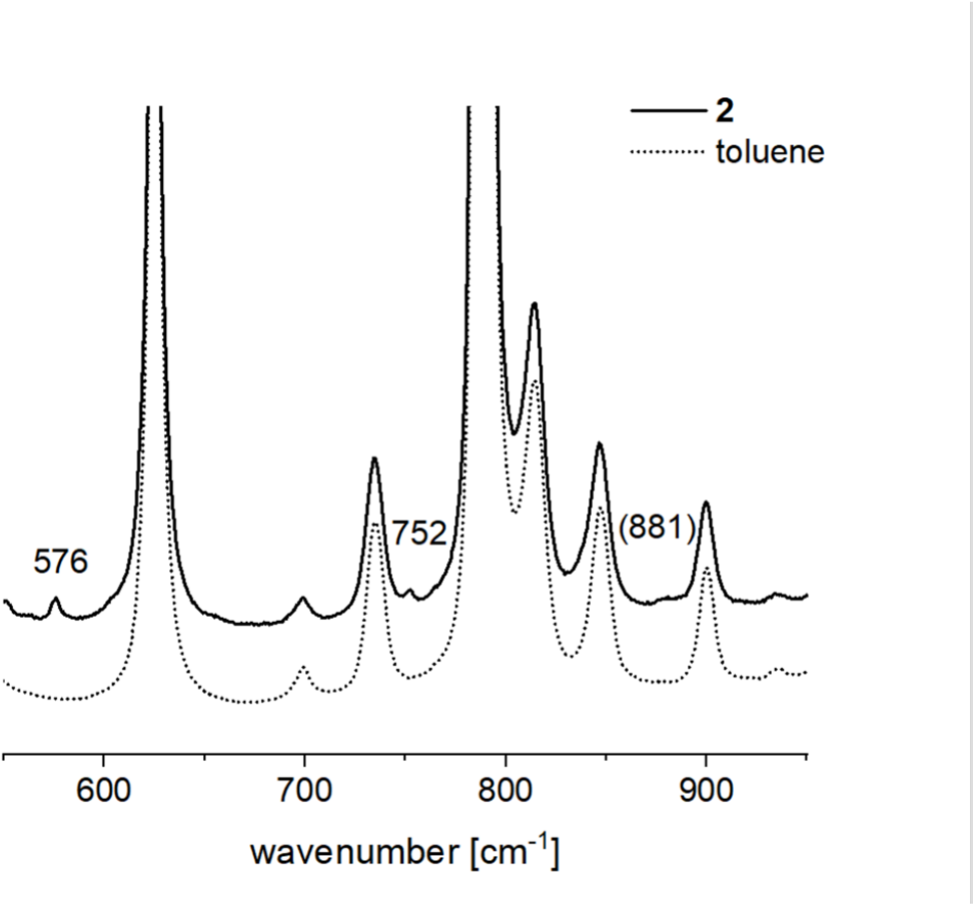
Resonance Raman spectrum recorded of [Tp^Mes^Fe(OO^*t*^Bu)(ACC)], **2**, dissolved in toluene using an excitation wavelength of 514 nm at – 60 °C (black, top) compared to the resonances of the solvent (gray, bottom)

The identification of the colored species as a high-spin iron(III) alkyl peroxide [Tp^Mes^Fe(OO^*t*^Bu)(ACC)], **2**, as shown in Scheme 7, was corroborated via resonance Raman spectroscopy (Fig. 6).

The resonance Raman spectrum of compound **2** exhibits two well-resolved, resonance-enhanced vibrational bands at 576 and 752 cm⁻^1^. A similar band near 580 cm⁻^1^ had previously been detected for compounds [Tp^Mes^Fe(OO^*t*^Bu)(OBz^*R*^)] [20], indicating a resonance-enhanced vibration in the Tp ligand. The band at 752 cm⁻^1^ was assigned to the C–O stretching vibration of the OOtBu ligand. The Fe–O stretching band was expected around 625 cm⁻^1^, likely obscured by a solvent band. The O–O stretching band is poorly resolved and can only be identified as a slight rise in the spectrum at 881 cm⁻^1^. Nevertheless, the positions of the C–O and O–O vibrational bands confirm that the species is a high-spin iron(III) alkylperoxide. The high-spin ground state of **2** was confirmed by EPR spectroscopy of a frozen toluene solution at 77 K, showing a characteristic signal at a g-value of 4.3 (Fig. S1).

To avoid consecutive reactions with excessive hydroperoxide, subsequent reactivity studies were conducted exclusively with two equivalents of ^*t*^BuOOH. First, in a UV–Vis experiment at – 80 °C, it was confirmed that the iron(III) alkylperoxide **2** can be fully generated even with just two equivalents of ^*t*^BuOOH (see SI and Fig. S3). Mechanistically, the first equivalent generates an iron(III) hydroxide with concomitant formation of ^*t*^BuO^.^, while the second equivalent leads to the formation of the FeOO^*t*^Bu unit and water [20].

**Fig. 7:**
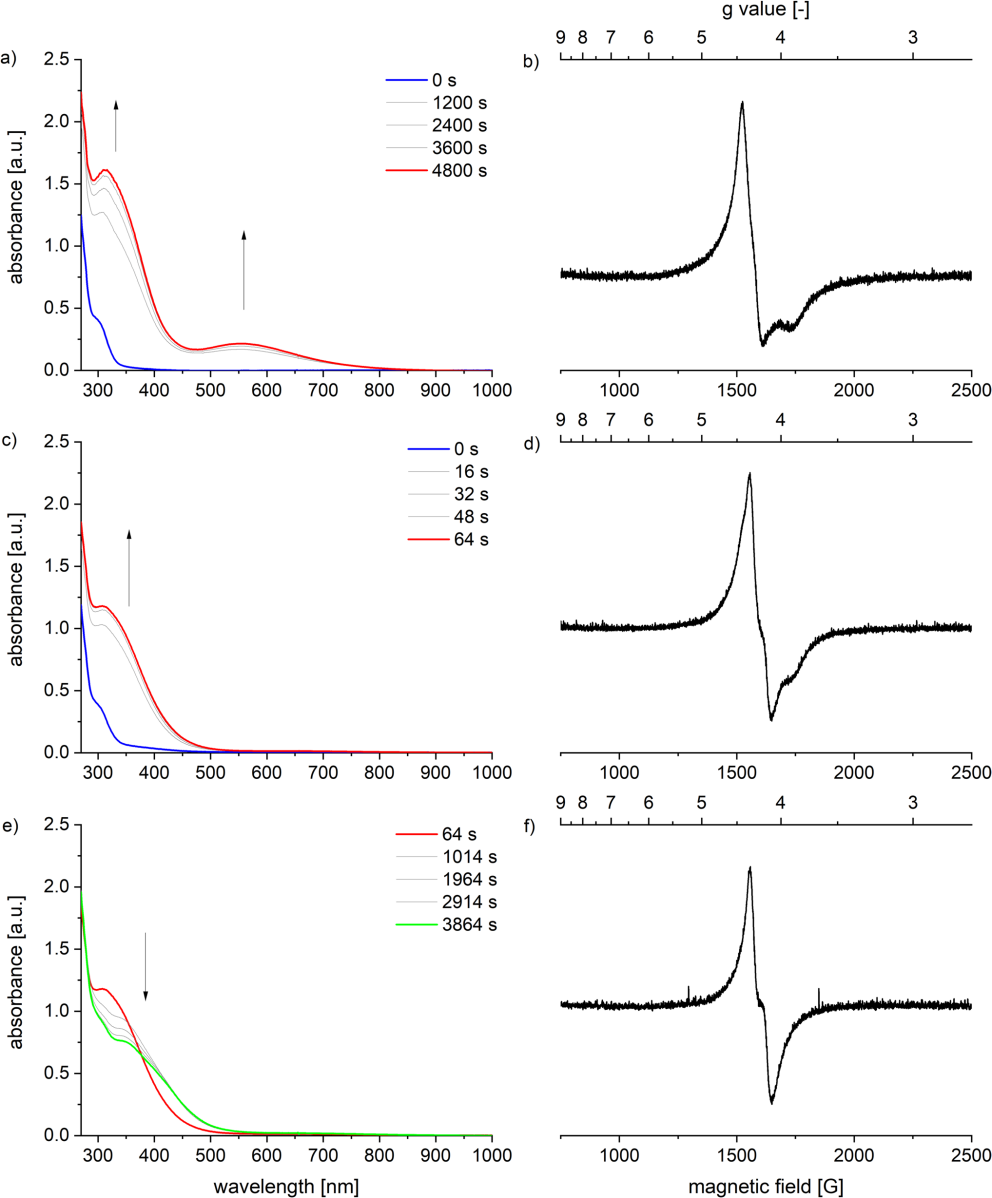
UV–Vis-spectra monitoring the reaction of [Tp^Mes^FeACC], **1**, with two equiv. ^*t*^BuOOH at **a** – 80 °C, 0—4800 s, **c** 25 °C, 0—64 s and **e** 25 °C 64—3864 s in DCM (0.4 mM) as well as EPR spectra of the same reaction at **b** – 60 °C, after 20 min, **d** after warming to RT and **f** after further 100 min. at RT in frozen toluene measured at 77 K (2 mM complex solution)

When compound **1** was reacted with ^*t*^BuOOH at room temperature, it did not form the high-spin iron(III) alkylperoxide. Instead, a different species was obtained, characterized by an absorption maximum at 315 nm but lacking the π* → *d*-CT band observed in the case of **2** (Fig. 7c). The UV–Vis spectrum of this species was identical to that of the decomposition product of **2**. Furthermore, an EPR spectrum confirmed that this species also has a high-spin ground state, indicated by a signal with a g-value of 4.3 (Fig. 7d). Over time, the absorbance at 315 nm decreased, and changes were noted in the EPR spectrum (Figs. 7e, f), suggesting that this species undergoes further decomposition in a consecutive reaction.

**Fig. 8:**
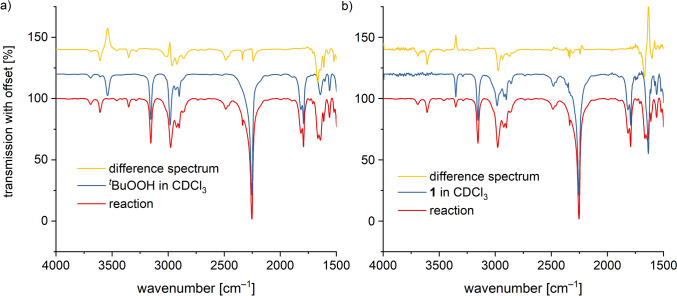
IR spectra of the reaction solution composed of [Tp^Mes^FeACC], **1**, with two equiv. of ^*t*^BuOOH (red) compared to the spectra of the reactants **a **^*t*^BuOOH in CDCl_3_ and **b** **1** in CDCl_3_ (blue) with the corresponding difference spectra (yellow) (room temperature, 20 mM complex)

To further investigate the reaction products, the reaction of **1** with ^*t*^BuOOH was carried out in deuterated chloroform (CDCl_3_), and IR spectra of the solutions were recorded using a CaF_2_ cell. For comparison, IR spectra of the starting compound **1**, ^*t*^BuOOH in CDCl_3_, and the pure solvent were taken. Changes in the vibrational bands during the reaction were identified using difference spectra (Fig. 8a, b). Due to strong absorption by the CaF_2_ crystal at low wavenumbers, the IR spectra were only analyzed in the range between 4000 and 1500 cm^–1^.

The difference between the pure solvent spectrum and that of ^*t*^BuOOH in CDCl_3_ revealed the presence of a small amount of water, indicated by a band at 3685 cm^–1^, alongside another band in the O–H stretching region at 3538 cm^–1^, which was assigned to the O–O–H vibration (data not shown). In the range between 2800 and 3000 cm^–1^, the C–H stretching bands of ^t^BuOOH were identified.

Comparing the IR spectrum of the reaction solution of **1** with ^*t*^BuOOH to that of ^*t*^BuOOH in CDCl_3_ showed the complete disappearance of the O–O–H band at 3538 cm^–1^, while a new band appeared at 3605 cm^–1^, corresponding to the O–H stretching vibration of *tert*-butanol (Fig. 8a). This indicates the complete conversion of the hydroperoxide. The position and intensity of the C–H stretching bands of the ^*t*^Bu group remained unchanged.

The difference spectrum of the spectra of **1** in CDCl_3_ and pure solvent shows all the characteristic vibrational bands that had already been observed in the ATR-IR spectrum (data not shown). The C=O vibration absorbs at 1629 cm^–1^, the B–H vibration at 2481 cm^–1^, the C–H vibrations between 2820 and 3050 cm^–1^, and the NH_2_ vibrations at 3283 and 3347 cm^–1^. When comparing the spectrum of **1** in CDCl_3_ with that of the reaction solution, it is evident that the bands of the carboxylate and amine have reduced intensity, while the C–H and B–H vibrational bands remain unchanged. This suggests that the Tp ligand remains intact, but the ACC substrate ligand has partially degraded. A new band appears at slightly higher wavenumbers as compared to the original carboxylate band, the origin of which, however, is unclear.

**Scheme 8:**
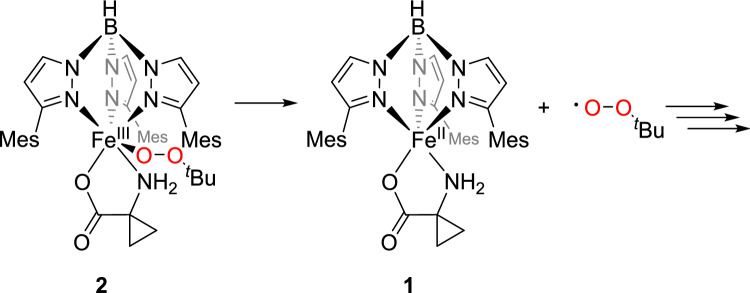
Proposed decomposition mechanism of the iron(III) alkyl peroxide complex [Tp^Mes^Fe(OO^*t*^Bu)(ACC)], **2**, via homolytic Fe–O bond cleavage followed by consecutive reactions with the resulting alkyl peroxide radicals

The incomplete conversion of the amino acid may indicate a degradation mechanism via a homolytic Fe–O bond cleavage. This would initially not significantly affect the amino acid ligand and would regenerate the starting complex **1**. Subsequently, an uncontrolled and incomplete reaction of the substrate ligand with the resulting peroxy radicals would be possible (Scheme 8). Consistent with this, there are no indications of degradation products such as ethylene, HCN, CO_2_, or water, which would have been detected through their characteristic vibrational bands.

In addition to the reaction solution, the gas phase above the solution was analyzed using IR spectroscopy and gas chromatography. No evidence of ethylene was found after the reaction, and only trace amounts of CO_2_ were detected.

The incomplete conversion of the substrate can be attributed to its reaction with released alkoxide and alkylperoxo radicals. Such a radical reaction with the substrate can be excluded for the enzyme, as it would be quickly deactivated by free radicals [1].

Thus, although the reaction of complex **1** with *tert*-butyl hydroperoxide can generate a high-spin iron(III) alkyl peroxide, this does not follow the biological decomposition mechanism. Instead of proceeding through a radical cascade leading to ethene, HCN, CO₂, and water, it undergoes an alternative, uncontrolled decomposition. In the preferred decomposition mechanism for high-spin iron(III) alkyl peroxides a homolytic Fe–O bond cleavage occurs in the initial step, and this is likely the case here as well. A homolytic O–O bond cleavage, which would generate a high-valent iron-oxo species as proposed in the mechanism by Klinman and colleagues, is unlikely to occur for **1**.[10]

### Reactivity towards mCPBA

**Scheme 9:**
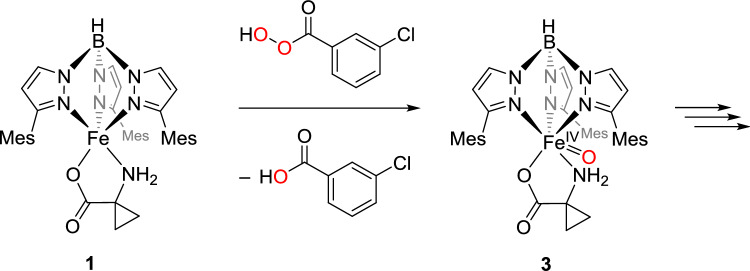
Proposed reaction pathway of [Tp^Mes^FeACC], **1**, with the OAT reagent mCPBA to give the iron(IV) oxo complex [Tp^Mes^Fe(ACC)(O)], **3**

According to the catalytic cycle proposed by Klinman and colleagues, another intermediate in the process is an iron(IV)-oxo species. Iron(II) complexes can be transformed into these highly reactive species using suitable oxygen transfer reagents such as iodosylbenzene (PhIO) or meta-chloroperbenzoic acid (mCPBA) [21, 22]. In previous experiments with [Tp^Ph,Me^FeACC], **III**, 55% ethylene were generated with an excess of PhIO and approximately 30% with mCPBA. It is noteworthy, though, that in control experiments using tetrabutylammonium-1-aminocyclopropanecarboxylate (Bu_4_NACC) and the corresponding oxidizing agents, larger amounts of ethylene were produced than with **III**.[13] For reactivity studies with compound **1**, mCPBA was chosen as the oxidizing agent because it has significantly better solubility in nonpolar solvents than PhIO, which provides advantages when applying a variety of spectroscopic methods. A possible reaction of **1** with mCPBA is illustrated in Scheme 9.

**Fig. 9:**
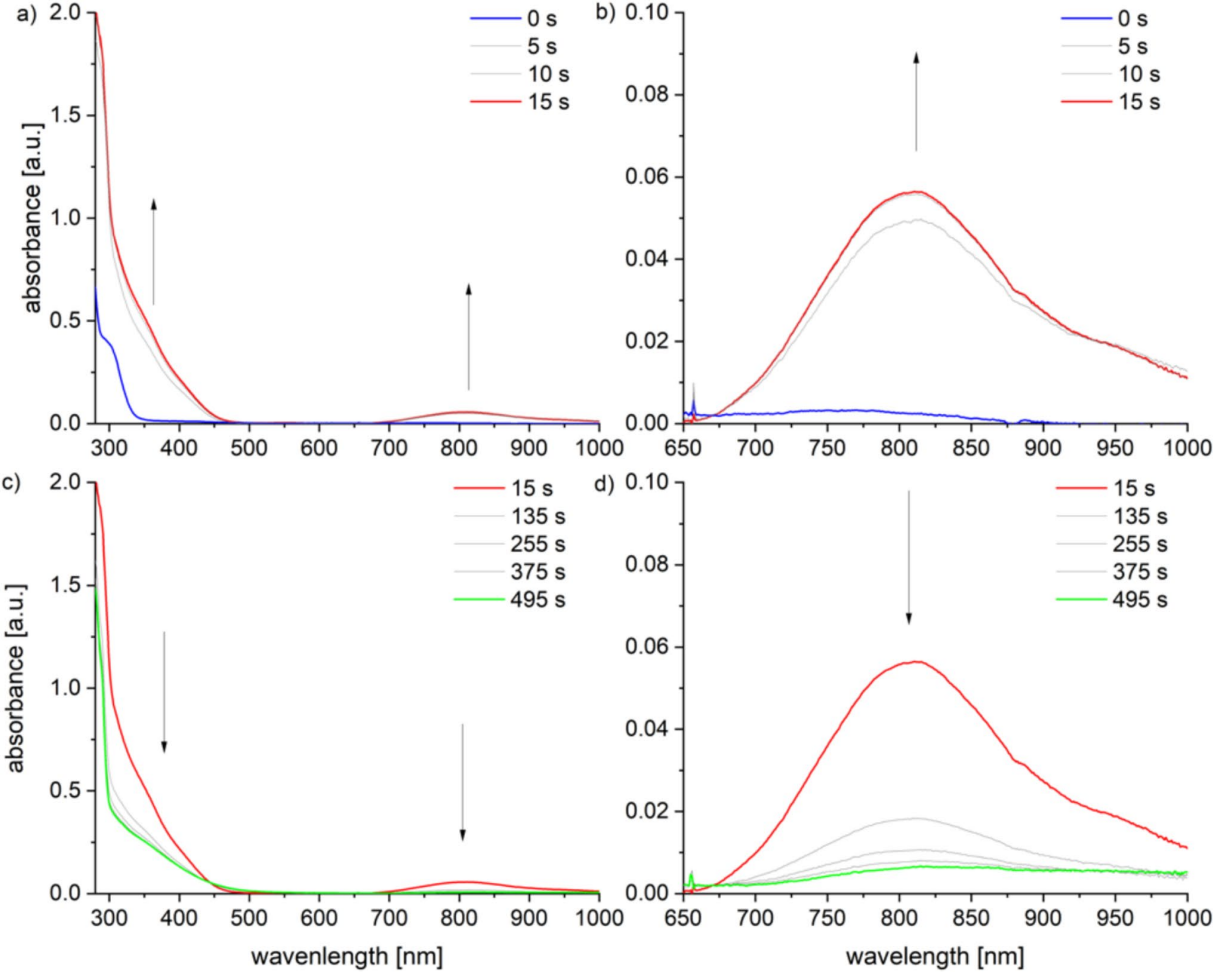
UV–Vis spectra a) and b) indicating the generation and c) and d) the decomposition of the metastable intermediate [Tp^Mes^Fe(ACC)(O)], **3**, generated from [Tp.^Mes^FeACC], **1**, and one equiv. of mCPBA in DCM at –80 °C (0.4 mM complex solution)

In the stoichiometric reaction with mCPBA, a weakly colored, metastable species, **3**, is formed at low temperatures. This species can be temporarily enriched and spectroscopically studied in a UV–Vis experiment at – 80 °C (Fig. 9). The formation occurs within 15 s, evidenced by an increase in absorbance between 280 and 450 nm (*ε*(350 nm) = 13,000 L·mol⁻^1^·cm⁻^1^) and a new band appearing at 810 nm (*ε*_max_(810 nm) = 1400 L·mol⁻^1^·cm⁻^1^). Due to its low stability and characteristic weak absorbance at 810 nm, the observed intermediate can be identified as an iron(IV) oxo species corresponding to [Tp^Mes^Fe(ACC)(O)], **3**, as depicted in Scheme 9.[21–23] The decomposition is manifested by the decrease in absorbance in the UV region of the spectrum and the complete disappearance of the band at 810 nm. The process was monitored at temperatures between 25 and – 90 °C, with the absorbance at 350 nm plotted logarithmically over time (Fig. S4), and it becomes obvious that the intermediate is only at temperatures below – 80 °C sufficiently stable.

The decomposition product from the room temperature reaction of compound **1** with one equivalent of mCPBA was analyzed using EPR spectroscopy in the X-band, in frozen toluene solution at 77 K. The spectrum obtained shortly after the reaction revealed a weak signal with a g-value of 4.25, which is characteristic of high-spin iron(III) complexes. However, in a second spectrum recorded after 100 min, this signal has disappeared entirely. This suggests that the decomposition product could be a spin-integer species that is EPR inactive in the X-band. It is clear that the species generated in this reaction does not match the decomposition product of **2** from the reaction with ^*t*^BuOOH.

Additionally, the reaction solution of **1** with mCPBA in CDCl_3_ was investigated by IR spectroscopy using a liquid IR cell (Figure S5). The transmission spectrum indicated that the Tp ligand had remained intact during the reaction, while all ACC signals had disappeared. However, no characteristic vibrational bands for expected decomposition products like ethene, HCN, CO_2_ or water were detected.

**Scheme 10:**
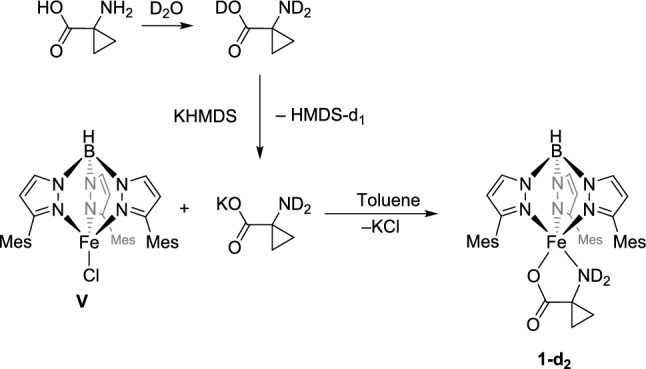
Synthesis of the doubly deuterated potassium salt of 1-aminocyclopropanecarboxylic acid, KACC-d_2_, and its subsequent reaction with the precursor complex** V** to yield [Tp.^Mes^FeACC-d_2_], **1-d**_**2**_

Attempts to identify further reaction products using gas chromatography, gas-phase IR, or NMR spectroscopy all failed. To investigate a possible kinetic isotope effect in the reaction, a complex deuterated at the amine function, [Tp^Mes^FeACC-d_2_], **1-d**_**2**_, was synthesized (Scheme 10). In the first step, 1-aminocyclopropanecarboxylic acid was dissolved in an excess of D_2_O. After a few minutes, all volatile components were completely removed under vacuum, and the deuterated amino acid ACCD-d_3_ was reacted with potassium hexamethyldisilazide. The deuterated potassium salt, KACC-d_2_, was then reacted with [Tp^Mes^FeCl], **V**, to produce **1-d**_**2**_.

The ^1^H-NMR spectrum of **1-d₂** is identical to that of **1**, which demonstrates that the NH₂ group’s signal in the spectrum of **1** is broadened into the baseline, likely due to its proximity to the iron atom. The ATR-IR spectrum of **1-d₂** shows only minor changes in the vibrational frequencies compared to **1**. All characteristic vibrational bands are present, confirming the successful synthesis and the expected κ^2^-N,O-binding mode of the amino acid. However, the vibrational bands of the amino group are significantly shifted. Instead of the NH₂ group’s signals at 3288 and 3353 cm⁻^1^, the absorption of the ND₂ group appears near the B–H stretching band at 2500 cm⁻^1^.

UV–Vis experiments with **1-d₂** under the same conditions as those with **1** show that the metastable intermediate **3-d₂** can also be enriched in a reaction with mCPBA at low temperatures. The decay rate of this species is identical to that of the non-deuterated species. The decrease in absorbance in the UV region and the band intensity at 810 nm occurs simultaneously at – 90 °C (Fig. S6). This indicates that there is no kinetic isotope effect, suggesting that an H atom abstraction from the amino group is not involved in the rate-determining step of the decomposition reaction (though ET/PT would still be feasible). Notably, for ACCO a rate-determining step involving either a proton-coupled electron transfer (PCET) or hydrogen atom transfer (HAT) has been suggested, which occurs during the formation of the Fe^IV^=O species and involves ascorbate [10].

A transmission infrared spectrum of the reaction solution of **1-d₂** with one equivalent of mCPBA in a CaF₂ liquid cell confirms, as was the case for **1**, the complete conversion of the substrate ligand, evidenced by the disappearance of the vibrational bands of the ND₂ and carboxylate groups (Fig. S7).

Paine and colleagues previously demonstrated that O_2_ reactions of Tp-iron-based model compounds with the non-native substrate ligands ACH and ADP proceed along an alternative pathway distinct from that of ACCO. The substrate ligands in [Tp^Ph₂^FeACH], **VI-b**, and [Tp^Ph₂^FeADP], **VI-c**, were converted to cyclohexanone and diphenyl ketone, respectively. However, experiments with ^18^O-labeled dioxygen and water were only partially helpful in elucidating the mechanism [14]. Without a clear characterization of the products from the reactions of **1** or **1-d₂** with mCPBA, discussing a mechanism here would be purely speculative. However, a reaction pathway involving a homolytic N–H bond cleavage, as suggested by Klinman for ACCO, can be ruled out for **1** due to the absence of a kinetic isotope effect.

### Cyclovoltammetry studies and in-situ O₂ activation

**Scheme 11:**
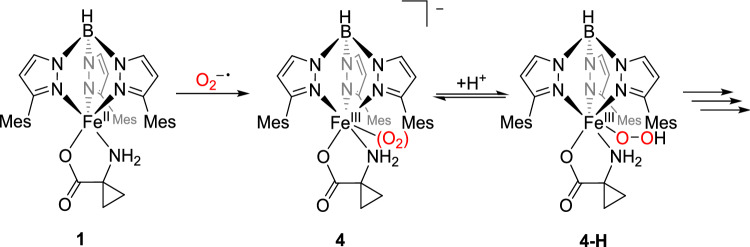
Proposed reaction of in-situ generated superoxide with [Tp^Mes^FeACC],** 1**, to give the peroxo- and hydroperoxo species **4** and **4-H**

Molecular oxygen can undergo up to four-electron electrochemical reduction, leading to products such as superoxide, hydrogen peroxide, hydroxyl radicals, and water (or exclusively water). The specific reduction potential depends on both the oxygen partial pressure and the solution’s pH [24]. For dry aprotic DMF, Oliveira et al. reported a reversible one-electron reduction with a value of – 0.85 V, measured against the standard calomel electrode [25]. The in-situ generated superoxide radical, O_2_^–•^, can react with divalent transition metal complexes, which reduce it to peroxide, leading to the formation of peroxo complexes [19, 26, 27]. If complex **1** reacted with in-situ generated superoxide analogously to the compounds described in the literature, this would also result in a peroxo complex, or a hydroperoxo complex upon protonation. The corresponding reaction scheme is shown in Scheme 11.

**Fig. 10:**
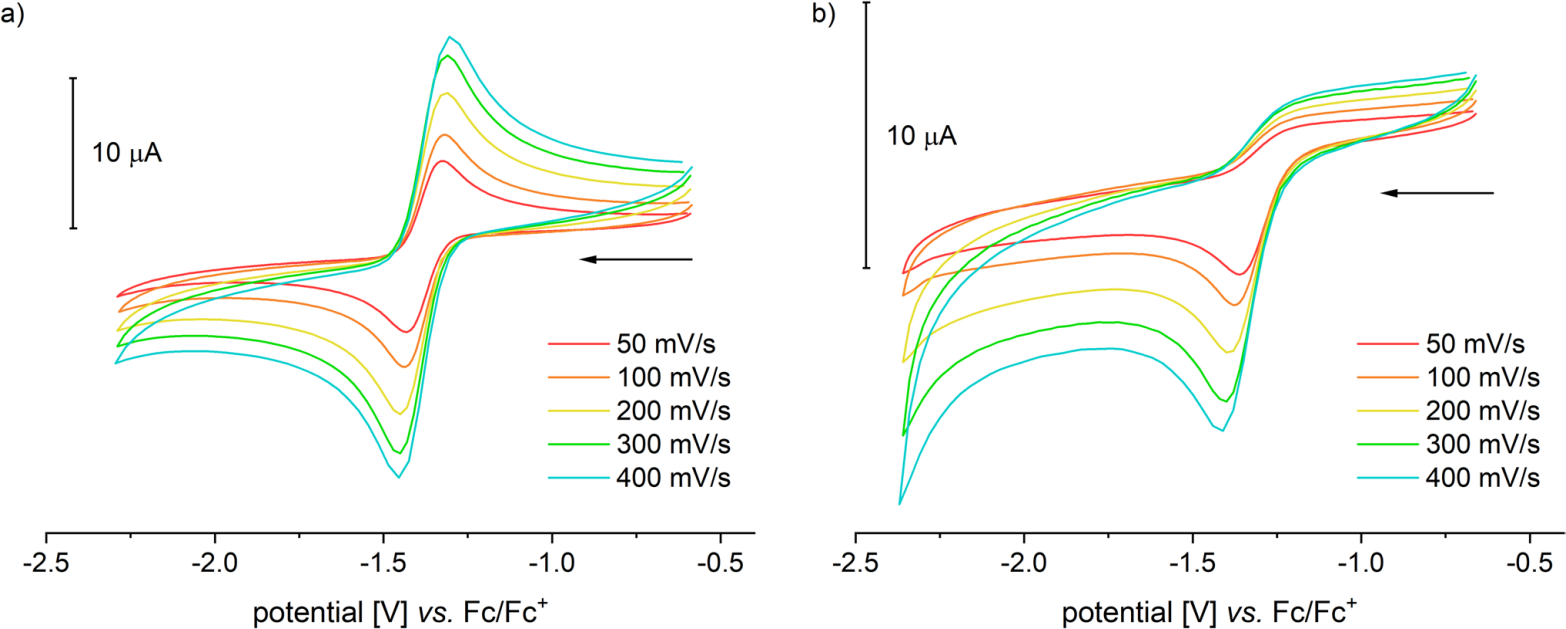
Cyclic voltammogram of **a** dioxygen in DMF and of **b** dioxygen in DMF in presence of [Tp.^Mes^FeACC], **1**, with scan rates between 50 and 400 mV/s (room temperature, 100 mM TBAPF, 1 mM complex)

A cyclic voltammogram of the first reversible reduction of dioxygen in DMF referenced against Fc/Fc^+^ is shown in Fig. 10a. The half wave potential E_1/2_ of –1,38 V is in good agreement with the value reported in the literature [28]. In the presence of complex **1** the dioxygen reduction becomes irreversible (Fig. 10b), as the in-situ generated superoxide radical reacts with compound **1**. The absence of a distinct plateau before oxygen reduction suggests that no intermediate oxygen adduct, **1**–O₂, is formed, which would otherwise be expected to reduce at a higher potential than oxygen itself [19, 29]. This is confirmed through cyclic voltammetry measurements conducted exclusively in the oxidative region, which demonstrate the reversible oxidation of complex **1**. The measurement in the presence of oxygen is identical to that without oxygen, and no color change occurs that would indicate a reaction between **1** and O₂. Therefore, the complex does not react with oxygen but does react with the superoxide radical.

**Fig. 11:**
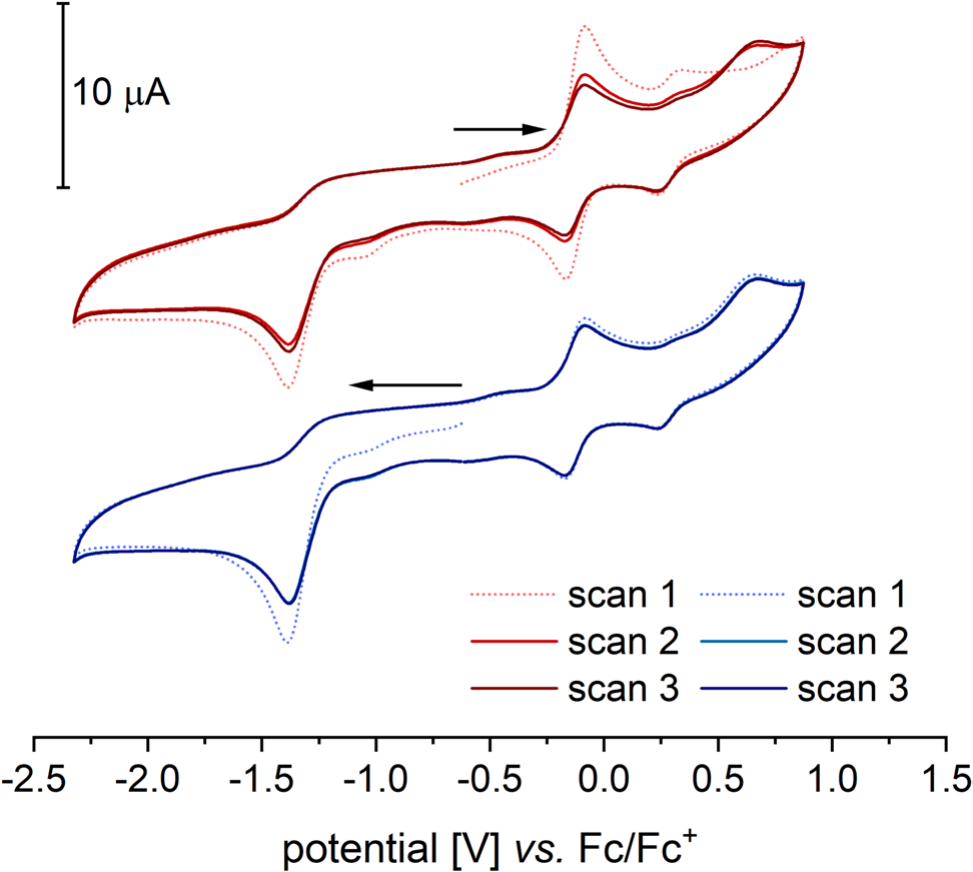
Reductive activation of O_2_ in the presence of [Tp.^Mes^FeACC], **1**, (room temperature, 0.1 V/s, 100 mM TBAPF, 1 mM complex)

When a cyclic voltammogram is measured over a broader range that includes both the oxidation of **1** and the reduction of oxygen, the resulting voltammograms depend on the measurement direction (Fig. 11). When starting with the oxidative region, the oxidation and subsequent reduction of **1** in the first cycle show no anomalies. The oxygen reduction remains irreversible, as before.

**Scheme 12:**
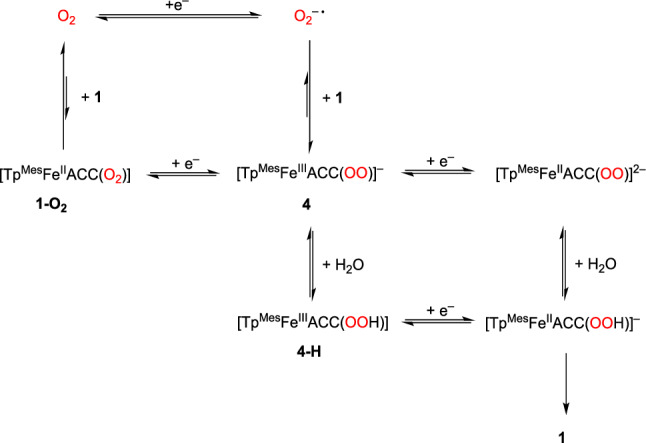
Proposed reduction mechanism in the reaction of dioxygen and [Tp^Mes^FeACC], **1**

However, in the second cycle, the oxidation current of **1** is significantly decreased, indicating consumption of the iron(II) species. In its place, an additional irreversible oxidation appears at 0.67 V. All further cycles are identical to this one. When the measurement begins in the reductive region followed by the oxidative, the first cycle of this voltammogram already resembles the second cycle in oxidizing direction. A pre-treatment by applying a negative voltage for 10 s has an identical effect on the cyclic voltammogram of **1**. Based on the electrochemical measurements, it can be assumed that initial reductive activation of O₂ takes place, followed by a reaction between the superoxide radical and complex **1**. The intermediate formation of an iron(III) peroxide, **4**, is plausible. In the presence of a proton source—such as residual moisture in the solvent—iron(III) peroxides exist in equilibrium with iron(III) hydroperoxides, as depicted for **4** and **4-H** in Scheme 12 [21, 30–34]. The detection of these (hydro)peroxide species via a consecutive reduction event is not possible, as they are typically reduced at higher potentials than oxygen [19]. The (hydro)peroxo complexes generated directly at the electrode are immediately reduced at a potential sufficient for oxygen reduction. Therefore, it is likely that the reduction of the intermediates contributes to the reduction current observed from –1.23 V onwards. As a result, accumulation of intermediates for spectroelectrochemical experiments appears highly improbable.

Ségaud et al. proposed that during the electrochemical reduction of [(TPEN)Fe(OO)]^+^, or [(TPEN)Fe(OOH)]^2+^ the initial complex [(TPEN)Fe]^2+^ with a divalent iron center is regenerated. Also, in this system, an irreversible oxidation emerged at a high potential following oxygen activation, attributed to the formation of iron(III) peroxide (0.74 V vs. SCE). The oxidation product might be an iron(IV) peroxo or iron(III) superoxo species, though the authors did not delve into this possibility further [19]. Bang et al. studied the side-on iron(III) peroxo complex [(TMC)Fe(O₂)]⁺, which exhibits an irreversible oxidation at 0.92 V and an irreversible reduction at –0.43 V (measured against the SCE). Chemical oxidation of this iron(III) peroxo complex releases oxygen, regenerating the corresponding iron(II) complex, [(TMC)Fe].^2^⁺ [35].

**Fig. 12:**
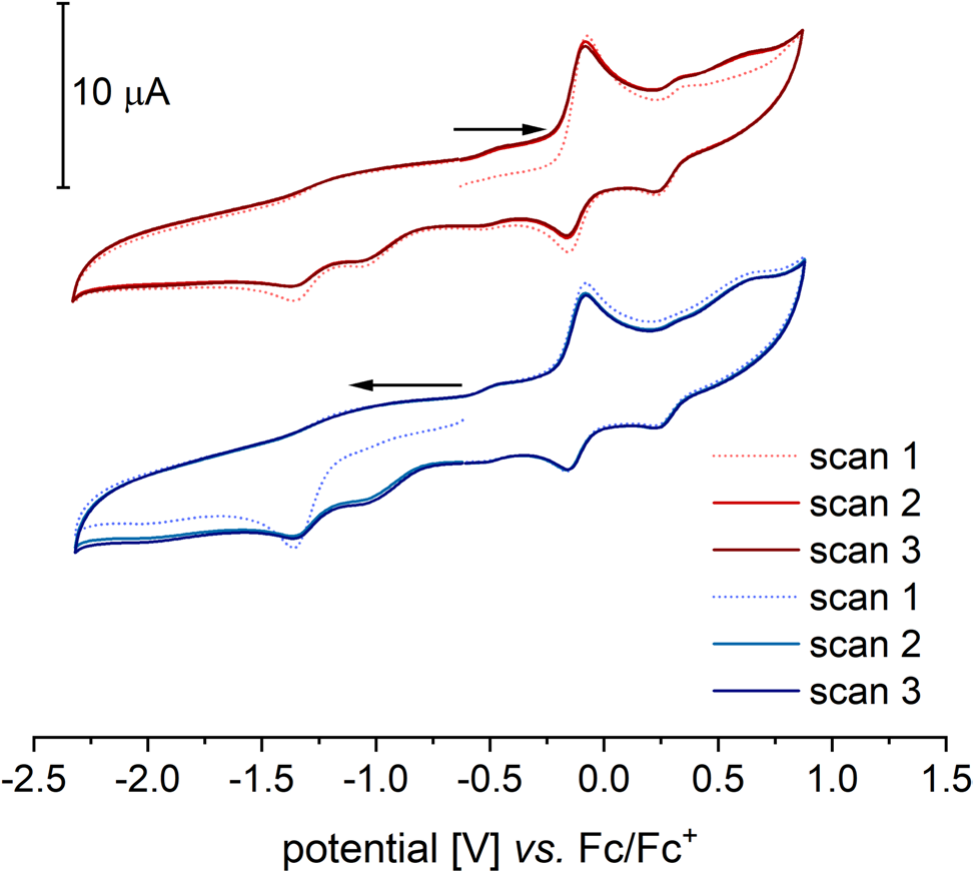
Reductive activation of O_2_ in the presence of [Tp.^Mes^FeACC], **1**, and 100 equivalents of water (room temperature, 0.1 V/s, 100 mM TBAPF, 1 mM complex)

Based on the observations of Ségaud et al. and Bang et al., the oxidation event observed at 0.67 V following the electrochemical activation of oxygen in the presence of complex **1** likely corresponds to the oxidation of the iron(III) peroxo species formed. Further evidence for the presence of an iron(III) peroxo species, **4**, is provided by the disappearance of the oxidation wave at 0.67 V upon the addition of a small amount of water (illustrated in Fig. 12). The equilibrium is shifted by the presence of the weak acid H₂O toward the formation of the corresponding hydroperoxide, **4-H**, and the oxidation of **4** is no longer observed. A similar effect was noted for [(TPEN)Fe(O₂)]⁺, whose oxidation wave completely disappeared upon water addition.

It was not possible to assign a definitive origin to the new irreversible cathodic wave at − 1.015 V that appears after adding water. Notably, this wave is coupled to a preceding oxidation event and occurs to the same extent even without additional oxygen.

Cyclic voltammetry measurements at low temperatures can help separate electrochemical processes from chemical decomposition events. This separation allows for the identification of intermediates when used in conjunction with other spectroscopic or spectrometric methods [36]. At low temperatures, the internal resistance of the electrolyte solution increases, which leads to a noticeable broadening of redox waves and greater separation of peak potentials. Additionally, the impact of the electrochemical double layer on the voltammogram becomes more pronounced [36, 37]. The comparison of the voltammograms at room temperature, 0, and – 45 °C clearly shows such broadening (Fig. S8).

Regardless, it is noteworthy that even at 0 °C, the current for the oxidation of **1** in the second cycle is barely smaller than in the first. Thus, the consumption of **1** is lower than at room temperature. Simultaneously, the current of the irreversible anodic event at 0.67 V, attributed to the oxidation of peroxide **4**, decreases significantly. This effect is even more pronounced at – 45 °C. At this temperature, complex **1** apparently is not consumed, and no oxidation occurs at 0.67 V anymore. Due to the slower diffusion of the intermediate superoxide and the iron(III) peroxide **4** at low temperatures, a consecutive reduction at the working electrode is more likely, and the starting compound **1** is fully recovered. A reaction scheme was developed based on the mechanism proposed for the [(TPEN)Fe].^2^⁺ system, as shown in Scheme 12 [19].

The individual steps that lead from the reduction of the (hydro-)peroxo complexes **4** and **4-H** back to the starting compound **1** are purely speculative at this point. It is possible that the release of hydrogen peroxide occurs through a heterolytic Fe–O bond cleavage or that an iron(IV) oxo species is formed through a heterolytic O–O bond breakage. The latter should be readily reducible at the cathode in the subsequent process. Both scenarios would be favored by the presence of a proton source [27].

## Conclusions

In summary, the introduction of bulky mesityl substituents at the 3-position of the Tp ligand enabled the synthesis and full characterization of a stable model complex for ACCO, namely [Tp^Mes^FeACC] (compound **1**). After reaction with *tert*-butyl hydroperoxide (tBuOOH) spectroscopic evidence for an alkylperoxido species was gained that may serve as a model for the reactive peroxo intermediate in ACCO’s catalytic cycle. This high-spin iron(III) alkyl peroxide, compound **2**, surprisingly did not follow a reaction pathway similar to that observed in ACCO and did not yield the expected products (ethylene, HCN, CO₂ and water). Nevertheless, valuable spectroscopic comparison data were obtained.

Additionally, the use of the oxygen-atom transfer reagent mCPBA enabled the modeling of another potential ACCO intermediate, an iron(IV) oxo species represented by compound **3**. This species, however, exhibited only a short lifespan, and none of the characteristic ACCO products were detected among its decomposition products, underscoring the unique challenge of modeling ACCO.

The in-situ electrochemical activation of oxygen provided conclusive evidence of the reactivity of the generated superoxide radical with compound **1**. Further, evidence was obtained for the presence of iron(III) (hydro)peroxo compounds **4** and **4-H**, considered possible key intermediates in the ACCO mechanism.

## Supplementary Information

Below is the link to the electronic supplementary material.

Supplementary file1 (DOCX 477 kb)

## Data Availability

No datasets were generated or analyzed during the current study.
