# Supplementary material for: A tris(pyrazolyl)-based model for the aminocyclopropane carboxylic acid oxidase and its behavior towards oxidants
Source: J Biol Inorg Chem. 2025 Sep 12;30(6-8):511–28. doi: 10.1007/s00775-025-02125-w (PMC12675639; doi:10.1007/s00775-025-02125-w)
Supplement: Supplementary file 1 — Supplementary file1 (DOCX 477 kb) [file 775_2025_2125_MOESM1_ESM.docx]

**Supplementary Material**

A Tris(pyrazolyl)-based model for the aminocyclopropane carboxylic acid oxidase and its behavior towards oxidants

Lars Müller, Charikleia Tzatza, Santina Hoof, Christian Limberg*

Humboldt-Universität zu Berlin, Institut für Chemie, Brook-Taylor-Strasse 2, 12489 Berlin (Germany), christian.limberg@chemie.hu-berlin.de

A. Jalila Simaan

Aix Marseille Univ, Centrale Marseille, CNRS, iSm2 UMR 7313, 13397, Marseille, (France)

**Experimental Section**

**Single-Crystal X-ray Diffraction**

Data collection was conducted using a Bruker D8 Venture or STOE IPDS 2 Θ diffractometer equipped with a flat-panel detector and Mo Kα radiation (λ = 0.71073 Å). Multi-scan absorption corrections were applied using SADABS. Structural solutions were determined using the intrinsic phasing method (SHELXT-2014)[1] and with the *full matrix least square procedures* based on F^2^ with all measured reflexes (Shelxl-2013 or Shelxl-2018 with the graphical interface SHELXle)[2] and anisotropic temperature factors for non-hydrogen atoms refined, with the exception of solvent molecules in compound **1**. All hydrogen atoms were calculated geometrically and refined using a riding model, with the exception of those bound to boron or nitrogen atoms, which were located in the residual electron density map and refined freely. The program Platon was used to apply the Squeeze procedure.[3] CCDC 2422686.

### **Nuclear magnetic resonance, NMR**

NMR spectra were recorded at room temperature using either a Bruker Avance DPX 300 or a Bruker Avance III 500 NMR spectrometer. Chemical shifts (δ) are given in ppm and coupling constants (J) in Hz. Proton signals are marked according to their splitting pattern, with "s" for singlet, "d" for doublet, "t" for triplet, and "m" for multiplet. Broadened signals are indicated with "br." Referencing was done relative to tetramethylsilane (TMS), with calibration based on residual proton signals from the solvent used in each case (C_6_D_6_: *δ* = 7.16 ppm, CDCl_3_: 7.27 ppm, CD_3_CN: 1.94 ppm, (CD_3_)_2_SO = 2.50 ppm, (CD_3_)_2_CO = 2.05 ppm).[4] In assigning signal designations, "Mes*" refers to the mesityl group attached at the 5-position of the pyrazole ring, while "Pz*" represents the pyrazolyl donor with a mesityl group also in the 5-position.

**High-Resolution Electrospray Ionization Mass Spectrometry (ESI-MS):**
High-resolution mass spectra were recorded using an Agilent Technologies 6210 Time-of-Flight LC-MS instrument equipped with an electrospray ionization (ESI) source. Acetonitrile was typically used as the eluent, but benzene, toluene, or dichloromethane was used when solubility in acetonitrile was insufficient.

**Attenuated Total Reflectance Infrared Spectroscopy (ATR-IR):**
ATR-IR spectra were collected from solid samples using a Bruker Alpha FTIR spectrometer, covering the range from 4000 to 400 cm⁻¹. Data analysis was performed with Bruker's Opus software. Solid samples were pressed onto the diamond crystal with a stamp for optimal contact.

**Electron Paramagnetic Spectroscopy (EPR):**
EPR spectra were measured at 77 K using a Magnitech MiniScope MS 5000 benchtop spectrometer, and data analysis was carried out using OriginPro software by OriginLab Corporation. EPR spectra simulations were performed with the EasySpin 5.2.30 plugin for MATLAB.

**Elemental Analysis (EA):**
Microanalyses were conducted using a HEKAtech Euro EA 3000 analyzer, with solid samples placed in tin boats. Carbon values for transition metal-hydrotris(pyrazolyl)borate complexes were already previously often found to be lower than theoretical values, while all other measurements closely match calculated values.[5]

**UV-Vis Spectroscopy**
UV-Vis spectra were recorded at variable temperatures using an Agilent 8453 UV-Vis spectrometer equipped with a Unisoku USP-203-A cryostat. SUPRASIL quartz cuvettes from Hellma Analytics with a 10 mm diameter were used for measurements. Solid-state UV-Vis spectra were acquired with a Cary 100 UV-Vis spectrometer (Agilent) from samples prepared as KBr pellets. Data analysis in both cases was performed using OriginPro software from OriginLab Corporation.

**Cyclic Voltammetry (CV)**
CV spectra were obtained with a PalmSens EmStat3 Blue instrument and BASi electrodes. The cell setup was consistent across measurements, using a glassy carbon disk as the working electrode, a platinum wire as the reference electrode, and a platinum disk as the counter electrode. Data analysis was conducted with PS Trace 5.5 software.

**^57^Fe Mössbauer Spectroscopy**
Mössbauer spectra of solid or dissolved samples were collected at 15 K using a SeeCo MS6 spectrometer. The sample was cooled with a Janis CCS-850 cryostat connected to a CTI-Cryogenic 8200 helium compressor, with temperature control managed by a LakeShore 335 module. Unless specified, samples were not enriched with ^57^Fe. Data analysis was conducted using WMOSS 4 software from wmoss.org.

**Starting Compounds and Reagents**
Solvents were dried and purified using a double-column argon-driven solvent purification system from MBraun. All chemicals were of at least reagent grade or higher and purchased commercially. Triethylamine was dried over calcium hydride under reflux and freshly distilled before use.

**Modified Synthesis of Potassium 1-Aminocyclopropanecarboxylate (KACC) and Potassium 1-Aminocyclopropanecarboxylate-d_2_ (KACC-d_2_) According to Sallmann et al**.[6]

For deuteration of the amino group in 1-aminocyclopropanecarboxylic acid (ACCH), the compound was dissolved minimally in D₂O, stirred for several hours, and then all volatile components were removed under vacuum. ACCD-d₂ was obtained quantitatively and used without further purification.

ACCH (202.2 mg, 2.00 mmol) or ACCD-d₂ (89.7 mg, 0.86 mmol) were dissolved in THF along with an equimolar amount of potassium hexamethyldisilazide (399.0 mg, 2.00 mmol; 171.7 mg, 0.86 mmol) and stirred overnight. The following day, all volatile components were removed under vacuum, with no further purification.

*KACC*

Yield: 139 mg (1.85 mmol, 93 %); ATR-IR: 3365, 3339 [ν(NH_2_)], 3279 [ν(C−H)], 1661 [ν(C=O)], ^1^H-NMR (300 MHz, (CD_3_)_2_SO): *δ* = 0.31 (dt, *J* = 3.5, 2.4 Hz, 2H, C*H*_2_), 0.77 (dt, *J* = 3.5, 2.4 Hz, 2H, C*H*_2_), 1.77 (s, br, 2H, N*H*_2_) ppm.

*KACC-d_2_*

Yield: 139 mg (0.79 mmol, 88 %); ATR-IR: 2940 [ν(C−H)], 2508, 2494 [ν(ND_2_)], 1661 [ν(C=O)], ^1^H-NMR (300 MHz, (CD_3_)_2_SO): *δ* = 0.31 (dd, *J* = 6.2, 3.8 Hz, 2H, C*H*_2_), 0.77 (dd, *J* = 6.2, 3.8 Hz, 2H, C*H*_2_) ppm.

### **Synthesis of [Tp^Mes^FeACC], 1**

Complex **V** (98.2 mg, 0.150 mmol) and potassium 1-aminocyclopropanecarboxylate (KACC, 25.1 mg, 0.180 mmol) were dispersed in toluene (15 mL). The mixture was stirred overnight and then filtered the next day. All volatile components were removed from the filtrate under vacuum, and the resulting solid was thoroughly washed with Et₂O and dried under vacuum.

Single crystals of **1**·0.5C₆D₆, suitable for structural elucidation via X-ray diffraction, were obtained by slow evaporation of solvent from a concentrated solution in benzene.

Yield: 80.5 mg (0,111 mmol, 74 %); Elemental analysis: calc. for C_40_H_46_BFeN_7_O_2_·0.5Tol (*MW* = 723.32 g/mol) C: 67.89, H: 6.55, N: 12.74 found C: 67.56, H: 6.65, N: 12.47; ATR-IR: 3353, 3288 [ν(NH_2_)], 2474 [ν(B−H)], 1658 [ν(C=O)] cm^–1^; ESI-MS (MeCN, pos): 427.1616 (38.1 %), 538.213 (58.3 %), 623.285 (59.0 %, [Tp^Mes^Fe]^+^), 724.341 (100 %, [M+H]^+^) m/z; ^1^H-NMR (300 MHz, C_6_D_6_): *δ* = –15.02 (s, 18H, *o*-MesC*H*_3_), 2.34 (s, 9H, *p*-MesC*H*_3_), 3.42 (s, 6H, *m*-MesC*H*), 23.86 (s, 2H, C*H*_2_), 30.08 (s, 1H, B*H*), 45.42 (s, 3H, 5-Pz*H*), 51.22 (s, 2H, C*H*_2_), 52.96 (s, 3H, 4-Pz*H*) ppm.

### **Synthesis of [Tp^Mes^FeACC-d_2_], 1-d_2_**

The synthesis followed the procedure described for [Tp^Mes^FeACC] using **V** (65.9 mg, 0.100 mmol)) and KACC-d_2_ (16.9 mg, 0.120 mmol) as precursors.

Yield: 55.3 mg (0.076 mmol, 76 %); ATR-IR: 2446 [ν(B−H)], 2516 [ν(ND_2_)], 1656 [ν(C=O)] cm^–1^; ^1^H-NMR (300 MHz, C_6_D_6_): *δ* = –15.15 (s, 18H, *o*-MesC*H*_3_), 2.34 (s, 9H, *p*-MesC*H*_3_), 3.40 (s, 6H, *m*-MesC*H*) 23.83 (s, 2H, C*H*_2_), 29.96 (s, 1H, B*H*), 45.72 (s, 3H, 5-Pz*H*), 51.57 (s, 2H, C*H*_2_), 53.26 (s, 3H, 4-Pz*H*) ppm.

**EPR spectra**


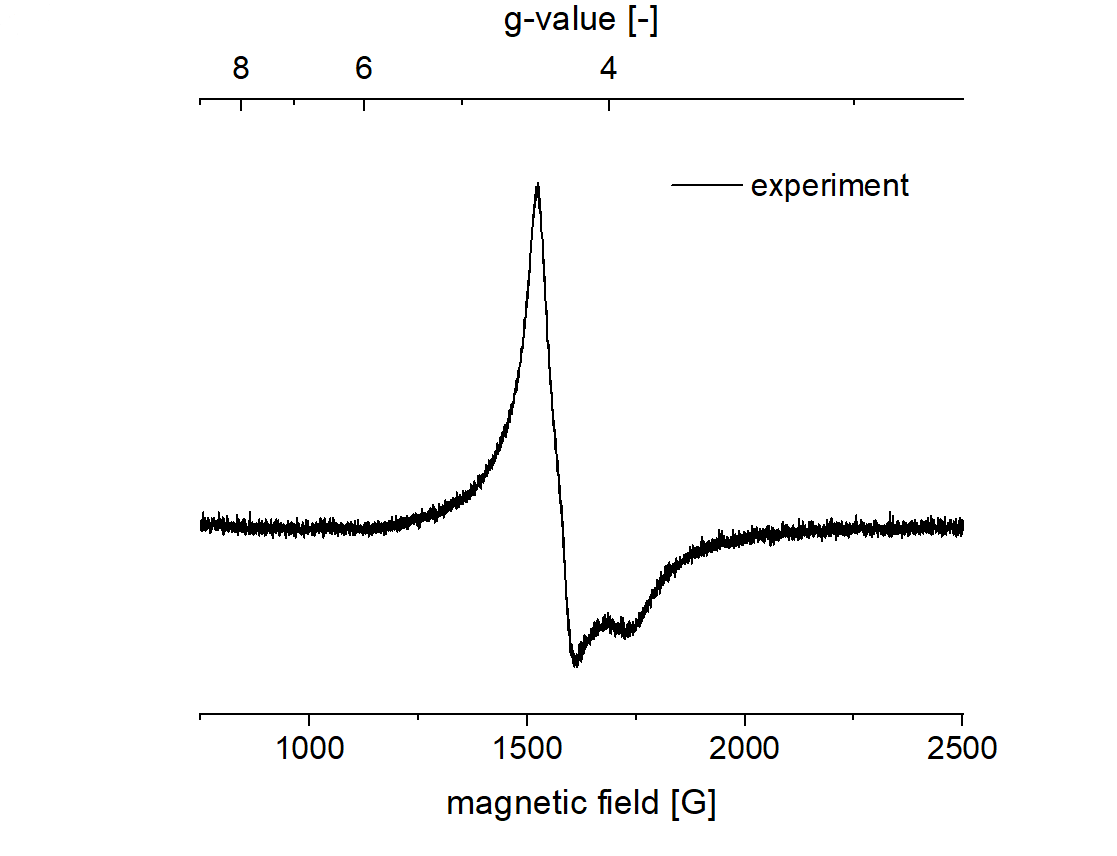


**Fig. S 1** ESR spectrum of the reaction mixture of [Tp^Mes^Fe(OO*^t^*Bu)(ACC)], **2**, in frozen toluene at 77 K, previously generated at –60 °C.

**Test of O_2_ reactivity**

Conditions: in DMF placed under air at 40°C in a sealed vial (180 microL). The complex was prepared anaerobically and 20 micoL added through the septum. Gas from the headspace was injected in GC (1 mL). Maximum conversion reached after ca. 20 minutes reaction.

**Fig. S 2** Production of ethylene from bound ACC in DMF at 40 °C. [Tp^Ph,Me^FeACC] = 0.1 mM.

### **Reactivity towards ^t^BuOOH**

Figure S3 shows the absorbances at 315 and 560 nm plotted against time after the addition of two or four equivalents of *^t^*BuOOH to a solution of **1** in DCM. While the reaction with a smaller excess of alkyl peroxide takes significantly longer, the same absorbances of 1.65 at 315 nm and 0.25 at 560 nm are achieved (ε_max_(315 nm) = 4125 L·mol^–1^·cm^–1^, ε_max_(560 nm) = 625 L·mol^–1^·cm^–1^). Additionally, the UV-Vis spectra obtained when using two and four equivalents of *^t^*BuOOH match.


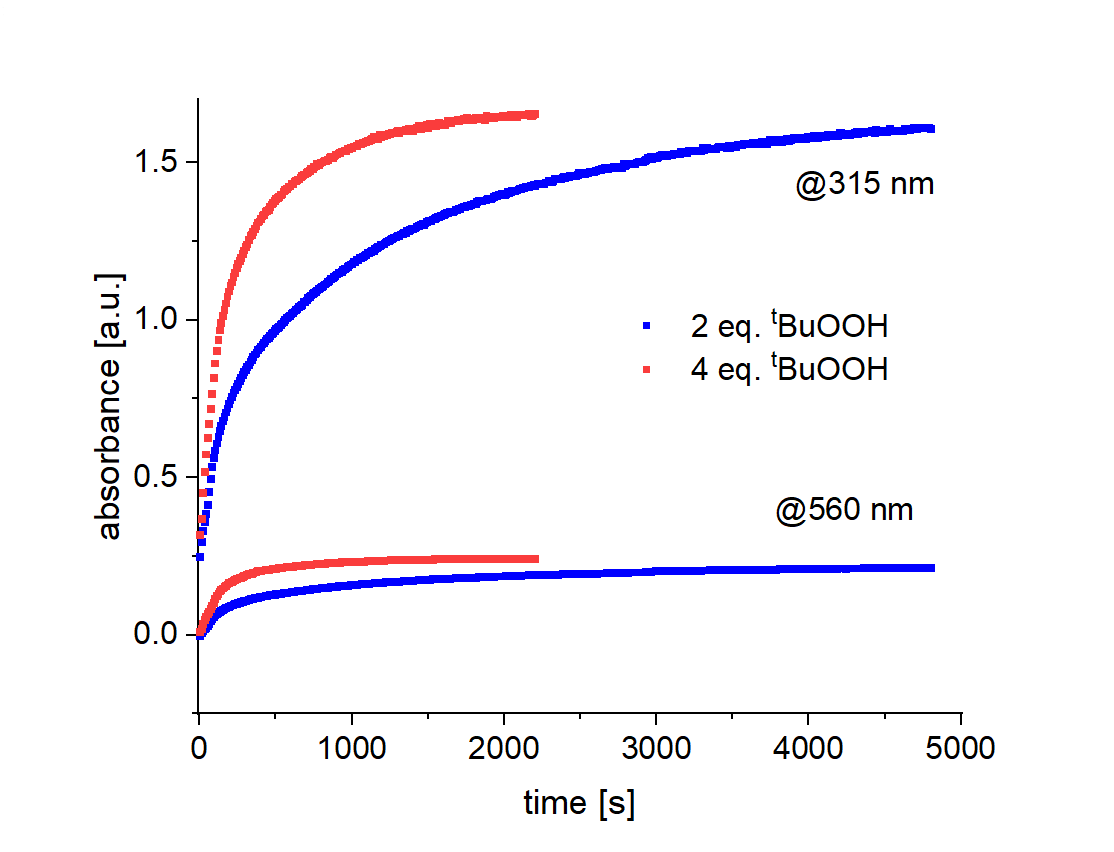


**Fig. S3** Absorbances at 315 und 560 nm plotted against reaction time of [Tp^Mes^FeACC)], **1**, with two (grey) and four (black) equiv. of *^t^*BuOOH, respectively, at –80 °C in DCM (0.4 mM)

### **Reactivity towards mCPBA**

The decomposition process of **3** was monitored at temperatures between 25 °C and –90 °C, with the absorbance at 350 nm plotted logarithmically over time (Figure S4). It becomes clear that the intermediate is only at temperatures below –80 °C sufficiently stable, and decomposition products are expected to appear in the reaction solution after a short period, regardless. Furthermore, the logarithmic plot indicates that the observed decomposition does not follow first-order kinetics, as there is no linear trend in the graph. It is important to note that due to the simultaneous formation and decomposition reactions, no conclusive statement about the kinetics—either for formation or decomposition—can be made. At higher temperatures, the stability of intermediate **3** decreases. At room temperature, only the slightly yellow decomposition product can be observed.


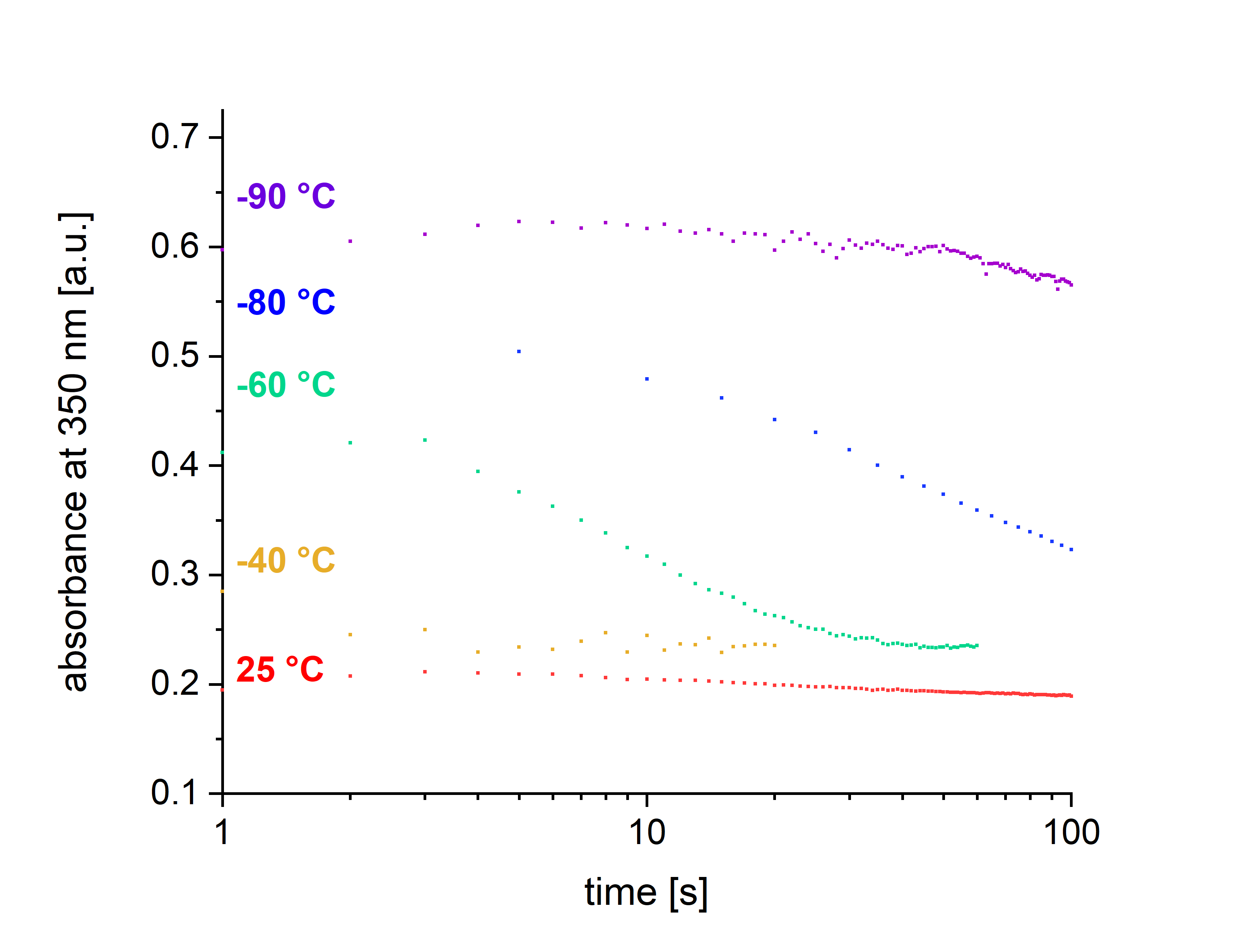


**Fig. S4** Time course of the decomposition of intermediate **3**, formed in the reaction of [Tp^Mes^FeACC], **1**, with mCPBA at temperatures between 25°C and –90°C in DCM (0.4 mM complex solution), plotted logarithmically against time

The transmission spectrum of the solution obtained in course of the reaction of **1** with mCPBA at r.t. (Figure S5) showed the characteristic C–H vibrations of the mesityl substituents between 2820 and 3050 cm^–1^, as well as the B–H vibration at 2481 cm^–1^, indicating that the Tp ligand had remained intact during the reaction. However, the signals corresponding to the ACC substrate ligand (the NH_2_ group at 3283 and 3347 cm^–1^, and the carboxylate group at 1629 cm^–1^) had almost entirely disappeared. Despite this, no characteristic vibrational bands for expected decomposition products like ethene, HCN, CO_2_ or water were detected.


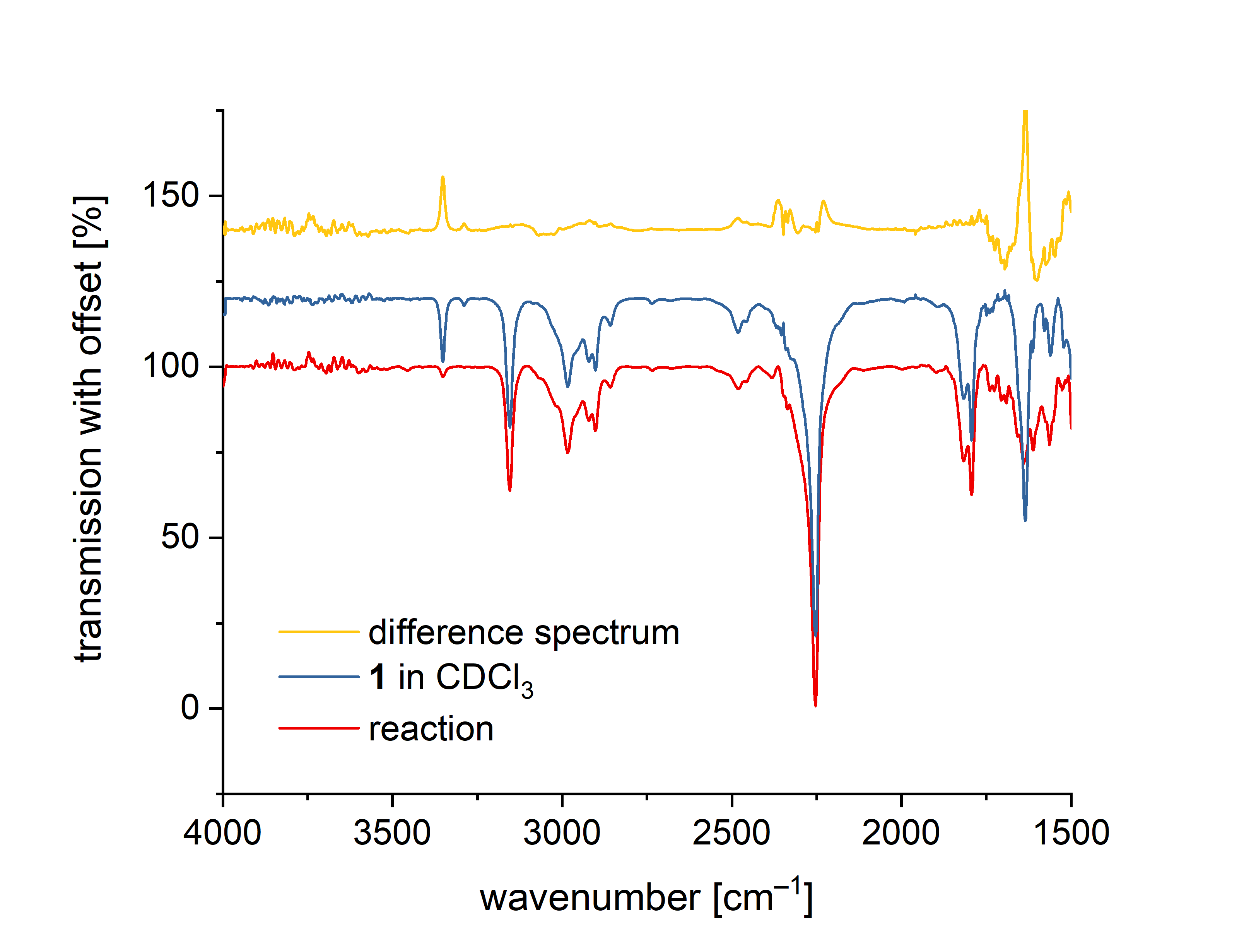


**Fig. S5** IR spectra of the solution obtained after reacting [Tp^Mes^FeACC], **1**, with one equiv. mCPBA (red) in comparison with the spectrum of **1** dissolved in CDCl_3_ (blue), and the corresponding difference spectrum (yellow) (room temperature, 20 mM complex)


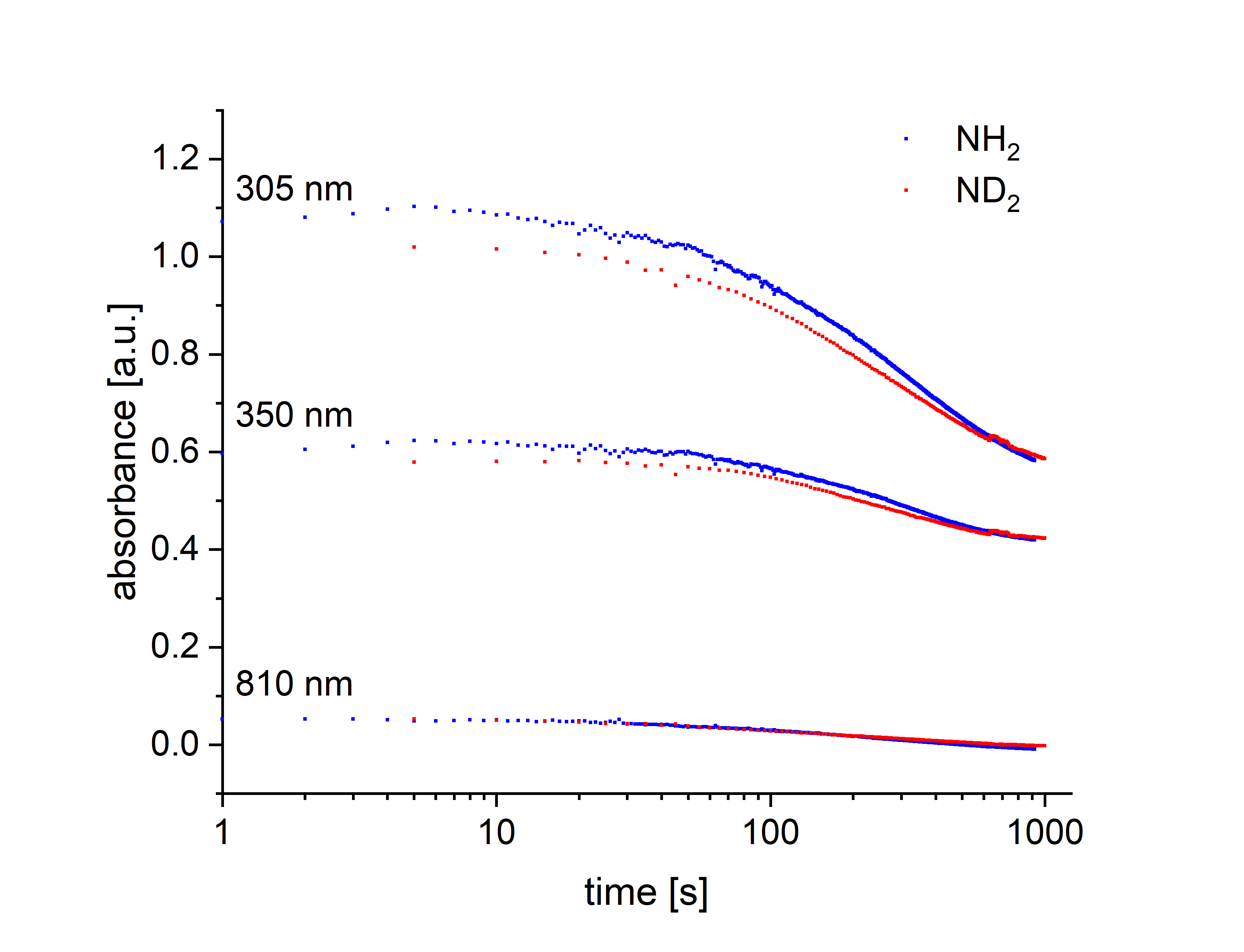


**Figure S6** Decrease of the bands absorbing at 305, 350 and 810 nm in the UV-Vis spectra of **1** and **1-d_2_** after treatment with one equiv. of mCPBA in DCM at –90 °C (0.4 mM complex)


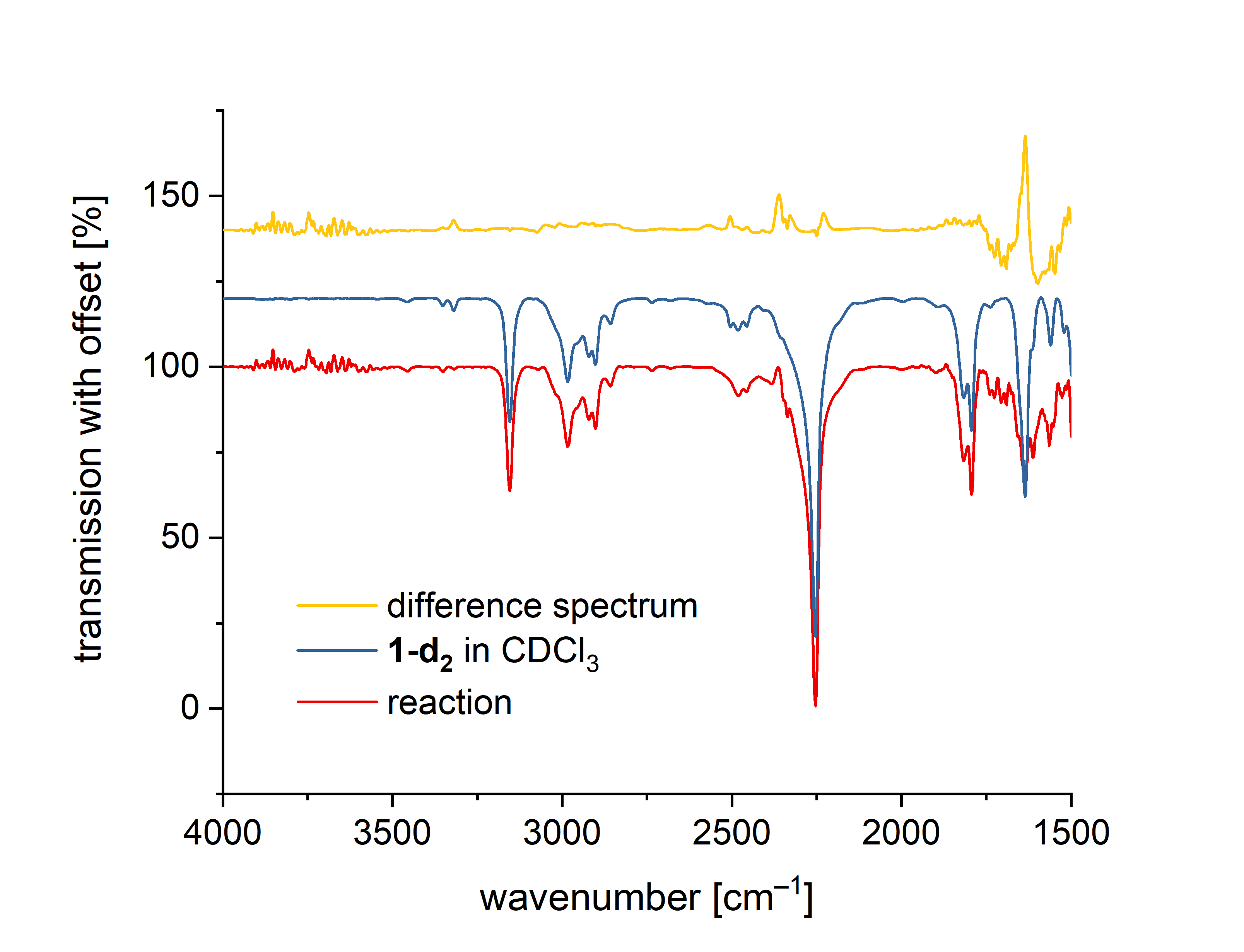


**Fig. S7** IR spectrum of the solution resulting from the reaction of [Tp^Mes^FeACC-d_2_], **1-d_2_**, with one equiv. of mCPBA (red) in comparison with the spectrum of **1-d_2_** dissolved in CDCl_3_ (blue), and the corresponding difference spectrum (yellow) (room temperature, 20 mM complex)


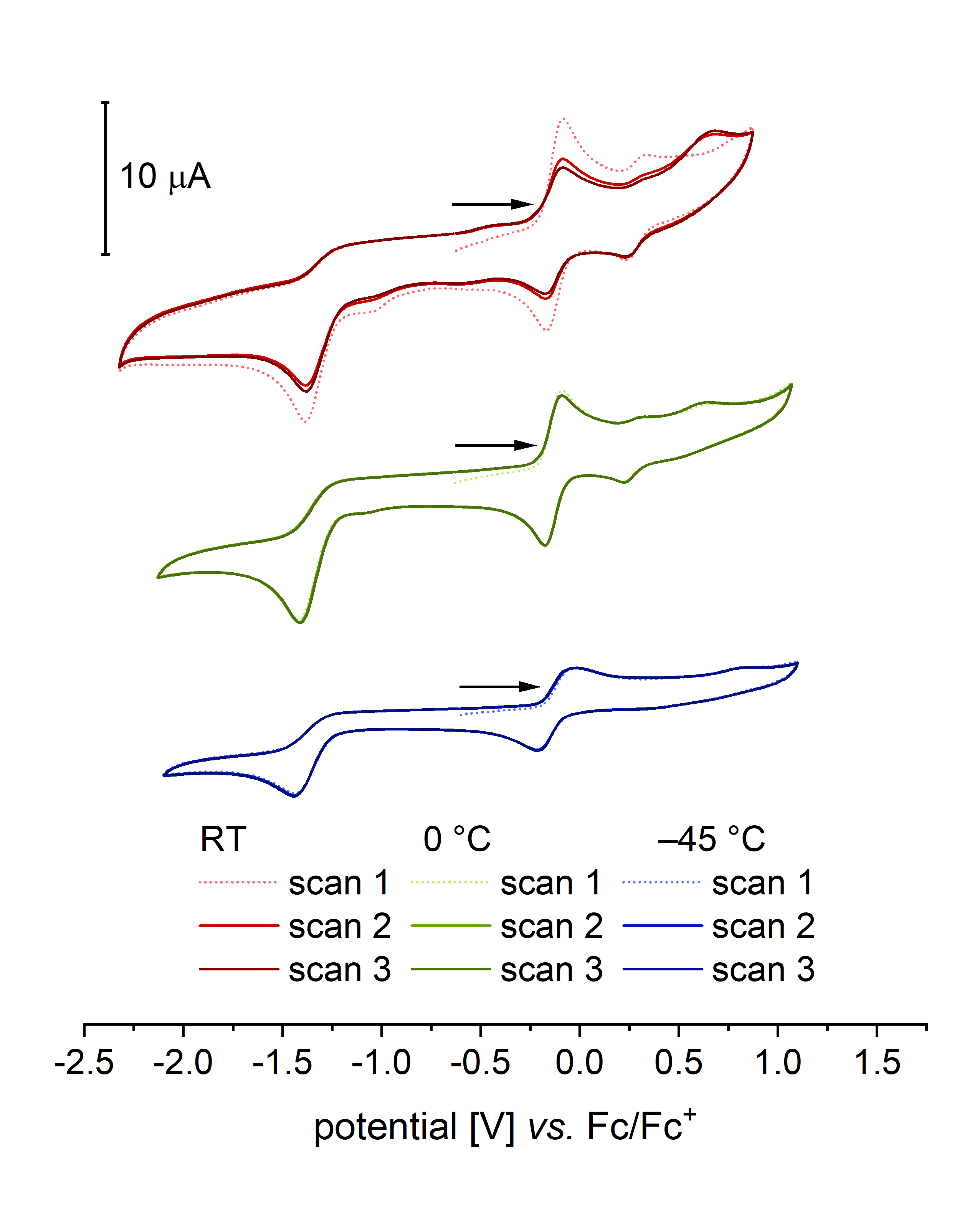


**Fig. S8** Reductive activation of O_2_ in the presence of [Tp^Mes^FeACC], **1**, at different temperatures: room temperature, 0 °C and –45 °C (0.1 V/s, 100 mM TBAPF, 1 mM complex)

Crystallographic data of [Tp^Mes^FeACC], **1**·0,5C_6_H_6_.

|  | **1**·0,5C_6_H_6_ |
| --- | --- |
| Empiric formula | C_43_H_49_BFeN_7_O_2_ |
| molar mass [g∙mol^–1^] | 762.55 |
| Crystal system | Orthorhombic |
| Crystal description | colorless block |
| Space group | *Pbca* |
| *a* [Å] | 16.4630(10) |
| *b* [Å] | 21.4086(13) |
| *c* [Å] | 22.4097(15) |
| *α* [°] | 90 |
| *β* [°] | 90 |
| *γ* [°] | 90 |
| *V* [Å^3^] | 7898.3(9) |
| *Z* | 8 |
| Berechn. Dichte [g∙cm^–3^] | 1.283 |
| *F(000)* | 3224 |
| Θ Weite [°] | 2.20-25.41 |
| measured reflexes | 197586 |
| independend reflexes | 7268 |
| refl. with *I*>2σ(*I*) | 6050 |
| *R*_int_ | 0.0862 |
| final *R* indices [*I*>2σ(*I*)] | R_1_ = 0.0366  *w*R_2_ = 0.0867 |
| *R* indices (all data) | R_1_ = 0.0484  *w*R_2_ = 0.0936 |
| GooF (all data) | 1.022 |
| completeness up to 25° | 0.999 |
| Min./max. residual electron density | -0.55 /+0.57 |

**References**

1. Sheldrick GM (2015) Crystal structure refinement with SHELXL. Acta Crystallogr Sect C 71:3–8. https://doi.org/10.1107/S2053229614024218

2. Hübschle CB, Sheldrick GM, Dittrich B (2011) ShelXle: a Qt graphical user interface for SHELXL. J Appl Crystallogr 44:1281–1284. https://doi.org/10.1107/S0021889811043202

3. Spek AL (2009) Structure validation in chemical crystallography. Acta Crystallogr Sect D 65:148–155. https://doi.org/10.1107/S090744490804362X

4. Fulmer GR, Miller AJM, Sherden NH, et al (2010) NMR Chemical Shifts of Trace Impurities : Common Laboratory Solvents , Organics , and Gases in Deuterated Solvents Relevant to the Organometallic Chemist. Organometallics 29:2176–2179. https://doi.org/10.1021/om100106e

5. Hoof S, Limberg C (2019) Bioinspired Trispyrazolylborato Nickel(II) Flavonolate Complexes and Their Reactivity Toward Dioxygen. Z Anorg Allg Chem 645:170–174. https://doi.org/10.1002/zaac.201800457

6. Sallmann M, Oldenburg F, Braun B, et al (2015) A Structural and Functional Model for the 1-Aminocyclopropane-1-carboxylic Acid Oxidase. Angew Chem Int Ed 54:12325–12328. https://doi.org/10.1002/anie.201502529
